# Targeting vulnerable microcircuits in the ventral hippocampus of male transgenic mice to rescue Alzheimer-like social memory loss

**DOI:** 10.1186/s40779-024-00512-z

**Published:** 2024-03-11

**Authors:** Hui-Yang Lei, Gui-Lin Pi, Ting He, Rui Xiong, Jing-Ru Lv, Jia-Le Liu, Dong-Qin Wu, Meng-Zhu Li, Kun Shi, Shi-Hong Li, Na-Na Yu, Yang Gao, Hui-Ling Yu, Lin-Yu Wei, Xin Wang, Qiu-Zhi Zhou, Pei-Lin Zou, Jia-Yang Zhou, Ying-Zhou Liu, Nai-Ting Shen, Jie Yang, Dan Ke, Qun Wang, Gong-Ping Liu, Xi-Fei Yang, Jian-Zhi Wang, Ying Yang

**Affiliations:** 1 Department of Pathophysiology, School of Basic Medicine, Key Laboratory of Education Ministry of China/Hubei Province for Neurological Disorders, Tongji Medical College, Huazhong University of Science and Technology, Wuhan, 430030 China; 2 Department of Traditional Chinese Medicine, The Central Hospital of Wuhan, Tongji Medical College, Huazhong University of Science and Technology, Wuhan, 430014 China; 3 Key Laboratory of Modern Toxicology of Shenzhen, Shenzhen Center for Disease Control and Prevention, Shenzhen, 518055 Guangdong China; 4 Co-Innovation Center of Neuroregeneration, Nantong University, Nantong, 226000 Jiangsu China

**Keywords:** Alzheimer’s disease, Tau protein, Ventral hippocampus, Social memory, Ursolic acid, Transcription factor EB (TFEB)

## Abstract

**Background:**

Episodic memory loss is a prominent clinical manifestation of Alzheimer’s disease (AD), which is closely related to tau pathology and hippocampal impairment. Due to the heterogeneity of brain neurons, the specific roles of different brain neurons in terms of their sensitivity to tau accumulation and their contribution to AD-like social memory loss remain unclear. Therefore, further investigation is necessary.

**Methods:**

We investigated the effects of AD-like tau pathology by Tandem mass tag proteomic and phosphoproteomic analysis, social behavioural tests, hippocampal electrophysiology, immunofluorescence staining and in vivo optical fibre recording of GCaMP6f and iGABASnFR. Additionally, we utilized optogenetics and administered ursolic acid (UA) via oral gavage to examine the effects of these agents on social memory in mice.

**Results:**

The results of proteomic and phosphoproteomic analyses revealed the characteristics of ventral hippocampal CA1 (vCA1) under both physiological conditions and AD-like tau pathology. As tau progressively accumulated, vCA1, especially its excitatory and parvalbumin (PV) neurons, were fully filled with mislocated and phosphorylated tau (p-Tau). This finding was not observed for dorsal hippocampal CA1 (dCA1). The overexpression of human tau (hTau) in excitatory and PV neurons mimicked AD-like tau accumulation, significantly inhibited neuronal excitability and suppressed distinct discrimination-associated firings of these neurons within vCA1. Photoactivating excitatory and PV neurons in vCA1 at specific rhythms and time windows efficiently ameliorated tau-impaired social memory. Notably, 1 month of UA administration efficiently decreased tau accumulation via autophagy in a transcription factor EB (TFEB)-dependent manner and restored the vCA1 microcircuit to ameliorate tau-impaired social memory.

**Conclusion:**

This study elucidated distinct protein and phosphoprotein networks between dCA1 and vCA1 and highlighted the susceptibility of the vCA1 microcircuit to AD-like tau accumulation. Notably, our novel findings regarding the efficacy of UA in reducing tau load and targeting the vCA1 microcircuit may provide a promising strategy for treating AD in the future.

**Supplementary Information:**

The online version contains supplementary material available at 10.1186/s40779-024-00512-z.

## Background

Alzheimer’s disease (AD) is a highly prevalent neurodegenerative disorder that causes dementia in elderly people [1]. In daily life, AD patients often suffer from the dilemma of not being able to recall when, where and/or who they have encountered. A series of studies showed that these domains of episodic memory are precisely processed through distinct cortical pathways [2–4]. Object or content processing involves an anterior-temporal system of connected regions, including the perirhinal cortex, amygdala, inferior temporal and fusiform gyrus as well as the lateral orbitofrontal and ventral temporopolar cortex. In contrast, spatial-contextual information processing relies on the posterior-medial system [2, 5], which comprises the parahippocampal cortex, retrosplenial cortex, precuneus posterior cingulate cortex and ventromedial prefrontal cortex. Finally, all these cortical streams converge into the hippocampus along the hippocampal anterior–posterior axis, thus indicating the crucial role of the hippocampus in successful encoding and retrieval of episodic memories. However, it remains unclear which hippocampal subregion along the anterior–posterior axis experiences dysfunction in AD patients and contributes to episodic memory deficits.

The formation of neurofibrillary tangles (NFTs) via intracellular tau accumulation is one of the pathological hallmarks of AD [6–8]. Clinically, NFT is positively correlated with the severity of dementia [9–11]. According to an event-based modeling approach, early signs of tau accumulation were observed in the entorhinal cortex and Brodmann area 35, followed by the anterior and posterior hippocampus, Brodmann area 36, parahippocampal, middle-temporal, inferior-temporal, inferior-parietal and retrosplenial cortex, and, ultimately, the precuneus [12]. Initially, tau pathology appears to predominantly accumulate in anterior medial temporal lobe regions before progressing towards more posterior and lateral temporal regions. In addition to destabilizing microtubules [13, 14], the accumulation of tau has been shown to inhibit synaptic plasticity [15], impair neurogenesis [16, 17], disturb the cell cycle [18] and suppress autophagy [16, 19], forming a vicious cycle to persistently trigger hippocampal dysfunction. From a behavioural perspective, exogenous expression of human tau (hTau), which mimics hippocampal tau accumulation, significantly impairs spatial memory in mice [20, 21].

Although these general tau expression experiments demonstrated the toxicity of tau accumulation, certain issues remain unaddressed: (1) whether various hippocampal subregions and neuron types exhibit distinct susceptibilities to tau pathology; (2) whether and how differential impairments in episodic memory domains result from cell type-specific tau accumulation; and (3) how to target tau accumulation to rescue episodic memory disorders in AD patients. In the present study, we utilized optogenetic manipulation, electrophysiological recording, in vivo calcium recording, and behavioural tests to comprehensively address these issues.

## Materials and methods

### Animals

One hundred and sixty-nine C57BL/6 adult mice were purchased from Beijing Vital River Laboratory Animal Technology Co., Ltd. P301S and wild type (WT) mice (*n* = 2 for each type) were purchased from Wuhan Youdu Biotechnology Co., Ltd. P301L and WT mice (*n* = 15 for each type), 3xTg-AD and nontransgenic (nTg) mice (*n* = 12 for each type) were kindly donated by Dr. Xifei Yang (Laboratory of Modern Toxicology of Shenzhen, Shenzhen Center for Disease Control and Prevention, Shenzhen, China). A total of 124 parvalbumin (PV)-Cre mice were generous gifts from Prof. You Wan (School of Medicine, Tsinghua University, Beijing, China). Mouse genotyping was performed following protocols described in the relevant literature [22–24].

P301L tau transgenic pR5 mice were generated on a genetic background of C57BL/6 × DBA2F1 [25, 26]. The line was backcrossed to C57BL/6 J mice a minimum of 10 times to generate C57BL/6 J congenic colonies. PV-Cre mice, also known as PV-Cre knock-in (Pvalb-IRES-Cre) mice, are strains that express Cre recombinase specifically in PV-expressing neurons (Jackson Laboratory, stock number 017320). By backcrossing PV-Cre mice with C57BL/6 J inbred mice for at least 5 generations, a C57BL/6 J-congenic colony was established. The B6C3F1 and B6;129 backgrounds were used for the P301S and 3xTg-AD mice, respectively.

The hemizygote × hemizygote breeding strategy was used to generate P301L mice and their corresponding WT counterparts. For P301S mice, hemizygote × noncarrier pairs were used for breeding. Homozygote × homozygote mating was conducted to generate 3xTg-AD mice and PV-Cre mice. Therefore, B6C3F1/J, B6129SF2/J and C57BL/6 J served as controls for the P301S mice, 3xTg-AD mice, and PV-Cre mice, respectively. For more detailed information, please visit the following websites: https://www.jax.org/strain/008169, https://www.jax.org/strain/004807, and https://www.jax.org/strain/017320.

Four mice per cage were housed at steady temperature (24–25 °C) under a 12-h light/dark cycle with food and autoclaved water available ad libitum. The experiments were conducted during the light cycle. All animal experiments were approved by the Animal Care and Use Committee (Huazhong University of Science and Technology, Wuhan, China; IACUC No. 3322).

### Stereotactic injection

The adeno-associated virus 2/9 (AAV2/9) vector was packaged and provided by BrainVTA (Wuhan, China), Brain Case (Wuhan, China), and OBiO Technology (Shanghai, China). Mice were anaesthetized with 1% sodium pentobarbital, and their heads were fixed in a stereotaxic instrument. According to the brain atlas coordinates, two holes were made in the mouse skull for the ventral hippocampal CA1 (vCA1) brain region [anteroposterior (AP): −3.28 mm, mediolateral (ML): ± 3.3 mm, dorsoventral (DV): −4.6 mm] or dorsal hippocampal CA1 (dCA1) region (AP: −2.06 mm, ML: ± 1.5 mm, DV: −1.5 mm) after iodophor sterilization. The viruses (0.2–0.3 µl, detailed information in Additional file 1: Table S1) were injected into the vCA1 or dCA1 with a 10 µl microsyringe. Brain slices corresponding to the AP (−3.08 mm to −3.52 mm) layer were carefully chosen for PV staining and subsequent statistical analysis via cell counting. The needle was left for 10 min to allow the virus to spread slowly at the injection site. The viral titre ranged from 2.38 × 10^12^ vg/ml to 7.38 × 10^12^ vg/ml. AAV viruses were expressed for 4 weeks before being subjected to behavioural experiments. After the behavioural tests, the mice were sacrificed, and the infection sites were confirmed by examining the expression of mCherry or enhanced green fluorescent protein (eGFP). In the present study, 5 mice were excluded due to incorrect viral expression.

### Cell culture and cell transfection

#### Cell culture

Primary hippocampal neurons were cultured from Sprague–Dawley rat embryos aged E13-E15. The embryos were removed from pregnant rats, and the hippocampus was carefully isolated on ice. The tissues were then dissected in DMEM/F12 (SH30023.01, HyClone, USA) and subjected to 20 min of digestion in a 0.125% (vol/vol) trypsin solution at 37 °C. Foetal bovine serum was used to terminate the enzyme activity. Subsequently, the neurons were treated with DMEM/F12 containing 10% foetal bovine serum for 4–6 h, followed by culture in neurobasal medium (21103049, Gibco, USA) supplemented with 1% GlutaMAX, 2% B27, and 1% penicillin–streptomycin (15140122; Thermo Fisher Scientific, USA). The culture dishes were precoated with 100 μg/ml poly-D-lysine. Within 4 h, the culture medium was replaced, and the serum was removed. Forty-eight hours later, pLenti-hSyn-MAPT-eGFP-3xFLAG-WPRE was transiently transfected, and proteins were collected 1 week later.

#### Cell transfection

HEK293-hTau cells were transiently transfected with 1 µg of plasmid expressing mCherry red fluorescence protein-GFP-microtubule-associated protein 1A/1B light chain 3 (mRFP-GFP-LC3) using 2 µl of Neofect™ DNA Transfection Reagent (TF20121201, Neofector, China) according to the manufacturer’s protocol. The cells were incubated for 24 h after transfection.

### Cell viability analysis

The viability of HEK293 (RRID:CVCL_0045, Cat#: GDC0067, China Center for Type Culture Collection) cells was evaluated using a Cell Counting Kit-8 (CCK-8) according to the manufacturer’s instructions. Cells were seeded in a 96-well plate (8000 cells/well) and then treated with various concentrations of ursolic acid (UA; 1, 10, 20, 30, 40, 50 and 100 μmol/L) for 6, 12 and 24 h. After UA treatment, the culture medium was removed, and 10 µl of CCK-8 solution was added to 90 μl of culture medium. After incubating in an incubator at 37 °C for 30 min with 5% CO_2_, the light absorbance was measured at 450 nm using a microplate reader (250058, BioTek, China).

### Quantitative real-time polymerase chain reaction (PCR)

Total RNA was isolated by using a TRIzol™ kit (Invitrogen, Carlsbad, CA, USA), and cDNA was obtained using a reverse transcription kit (RR037, TaKaRa, China). Real-time PCR was performed using a StepOnePlus™ Real-Time PCR System (272001262, AB Applied Biosystems, Cossell Biotechnology, China). The PCR mixture contained 10 μl of SYBR Green PCR master mix, 7 μl of diethylpyrocarbonate (DEPC H_2_O), 2 μl of forward and reverse primers, and 1 μl of cDNA. The sequences of the forward and reverse primers used for microtubule-associated protein tau (MAPT) were 5′-CAGCTCCGGCACCAACAG-3′ and 5′-CCTGGTTCAAAGTTCACCTGAT-3′, respectively. The β-actin-forward and reverse primers used were 5′-GAGACCTTCAACACCCCAGC-3′ and 5′-GGAGAGCATAGCCCTCGTAGAT-3′, respectively.

### Patch clamp electrophysiology

The brain was quickly removed after intracardial perfusion with 4 °C cutting solution. The cutting solution contained 2.5 mmol/L KCl, 1 mmol/L NaH_2_PO_4_, 26 mmol/L NaHCO_3_, 11 mmol/L glucose, 228 mmol/L sucrose, 7 mmol/L MgSO_4_, and 0.5 mmol/L CaCl_2_ (280–290 mOsm, pH 7.2–7.3). Coronal sections. (300 µm) were cut by a vibratome (VT1000S, Leica, Germany) and transferred to artificial cerebrospinal fluid (aCSF), which contained 119 mmol/L NaCl, 2.5 mmol/L KCl, 1 mmol/L NaH_2_PO_4_, 26 mmol/L NaHCO_3_, 11 mmol/L glucose, 1.3 mmol/L MgSO_4_, and 2.5 mmol/L CaCl_2_ (280–290 mOsm, pH 7.3). The brain slices were incubated at 34 °C for 30 min and then at room temperature for 1 h prior to electrophysiological recordings. All the solutions were saturated with 95% O_2_ and 5% CO_2_. The recording electrode (4–7 MΩ) was filled with 140 mmol/L K-gluconate, 10 mmol/L KCL, 20 mmol/L HEPES, 3 mmol/L MgCl_2_, 10 mmol/L EGTA, 1 mmol/L CaCl_2_, 4 mmol/L MgATP, and 5 mmol/L NaGTP (290–300 mOsm, pH 7.3). The action potential and optically elicited excitatory postsynaptic currents were recorded. The data were recorded using Multiclamp 700B amplifiers (Molecular Devices, Sunnyvale, CA, USA) and Digidata 1550B digital-to-analogue converters. The analogue signals at a frequency of 10 kHz were captured using pClamp software 10 (Molecular Devices, Sunnyvale, CA, USA). For light stimulation, Master 9 software was used to control the light-emitting diode (LED) light source, which emits light at a wavelength of 473 nm for a duration of 5 ms. The action potential threshold was measured as the voltage at which the first derivative of the voltage (dV/dt) exceeded the threshold (20 mV/ms). The data analysis was carried out using Clampfit10 software (Molecular Devices, Sunnyvale, CA, USA).

### Behavioural tests

Adult male mice were used for all the behavioural tests. Mice were allowed to acclimate to the room environment for 1 h before the behavioural experiments. The background luminance was 15 lux, and the light sources were well distributed around the chamber to avoid the innate preference for darkness in mice. The environment was maintained in a quiet state. The movement speed and total distance were recorded by video recording-tracking software (Chengdu Taimeng Software Co., Ltd., Chengdu, China). The interaction time, also known as the time of investigation, was recorded and scored. Alcohol (75%) was used to eliminate odours during the experimental interval. Social exploration, familiarization and memory tests were also conducted on P301L, 3xTg-AD, dCA1-hTau, vCA1-hTau, vCA1-hTau^CaMKII^, vCA1-hTau^PV^ mice and their controls (*n* = 8–10 per group). A novel object recognition test was also conducted on vCA1-hTau and control mice (*n* = 8 per group).

#### Social exploration test

A mouse was placed in the 50 cm × 50 cm × 50 cm open field for 5 min with two empty cups placed on opposing corners of the arena. Next, a novel adult male C57BL/6 mouse was placed in one of the corner cups. The time spent exploring the social cup and the empty cup was analysed by an investigator who was blinded to the animals’ identity. Social exploration was defined by direct snout-to-cup contact, with at least two paws on the ground [27]. Time spent climbing on the chamber was not included in the analysis. The interaction time (%) was calculated as the exploration time/5 min.

#### Social familiarization and memory tests

The arena and devices used were the same as those used for the social exploration test. Mice were allowed to freely explore a novel adult male C57BL/6 mouse and an empty cup for 3 sessions, each lasting 5 min. During trials 1 to 3, subjects became familiar with the novel mouse. The intertrial interval between social familiarization trials was 10 min. The social memory test was conducted 10 min after the last social familiarization trial. In the social memory test, a novel mouse was placed in one of the cups, the familiar mouse was placed in the other, and the ability to distinguish between the two was tested. A realistic picture is provided in Additional file 1: Fig. S1. Behavioural tests were video-recorded using video recording-tracking software (Chengdu Taimeng Software Co., Ltd., Chengdu, China). Videos were manually scored to measure the duration of interaction with the novel and familiar mice. The investigator conducting the scoring was blinded to the animals’ identities. The discrimination score was calculated as the exploration time (novel mouse − familiar mouse)/(novel mouse + familiar mouse), i.e., (N − F)/(N + F).

#### Novel object recognition

Two identical rectangular objects labelled as object A were positioned in the centre of a 50 cm × 50 cm × 50 cm field arena, and the mice were given 10 min to familiarize themselves with these objects. Twenty-four hours later, object A was replaced with a triangular object called object B. Notable exploratory behaviour was defined as the animal’s snout being within 2 cm proximity to the object. Jumping on the object was excluded from the analysis. Video recordings were carefully examined and scored by researchers who were unaware of the object and animal identities. The discrimination score was calculated as the exploration time (B − A)/(B + A).

#### Elevated plus maze test

The maze comprises a central platform (6 cm × 6 cm) with two opposing closed arms (6 cm × 66 cm) and two open arms. The maze was elevated 50 cm above the floor. Each mouse was positioned on the central platform, facing an open arm, and allowed to freely explore the entire maze for 5 min. Video recording-tracking software (Chengdu Taimeng Software Co., Ltd., Chengdu, China) was used to quantify the time spent in the open arms and the number of entries into the open arms.

#### Open field test

The open field test was conducted using an opaque plastic container (50 cm × 50 cm × 50 cm). The central zone area, comprising 50% of the open field arena, was defined. Each mouse was placed in the central area and allowed to freely explore the field arena for 5 min. Computer software was used to automatically record the time spent in the central area as well as the distance travelled by the mice.

### In vivo optical fibre recording

To examine the changes in neuronal activity and γ-aminobutyric acid (GABA) release during behavioural tasks, vCA1-hTau^CaMKII^, vCA1-hTau^PV^, and control mice were used (*n* = 5–8 per group), and fluorescent signals from genetically encoded calcium indicators (GCaMP6f) and intensity-based GABA-sensing fluorescence reporters (iGABASnFR) [28, 29] were recorded using an optical fibre recording system (Thinker Tech Nanjing Biotech Limited Co., Ltd., Nanjing, China). After 3 weeks of injecting a mixture of viruses into the vCA1, an optic fibre cannula (diameter = 1.25 mm, NA = 0.37; Newdoon, China) was inserted through the cranium. The mice were individually housed for 1 week to allow for recovery. A 473 nm laser (0.01–0.02 mW) was applied using an optical fibre recording system, and the resulting fluorescent signals were recorded [30]. The original signal was demodulated and converted to dF/F through data analysis. dF/F was calculated as dF/F (%) = (F–F_0_)/(F_0_–F_offset_) × 100% [31]. Normalized dF/F measurements were used to monitor activity alterations in excitatory or PV neurons in the vCA1. Motion tracking and manual tagging were employed to mark the position of the mice during the social memory test. The eGFP group was used to correct motion artefacts. MATLAB 2017a (Huazhong University of Science and Technology, China) was used for the data analysis.

### Optogenetic manipulation in free-moving mice

In the social recognition test, optogenetic manipulation was conducted on vCA1-hTau^CaMKII^-channelrhodopsins-2 (ChR2), vCA1-hTau^PV^-ChR2 and control mice (*n* = 8–10 per group). First, a mixture of viruses was delivered into the vCA1 (AP: −3.28 mm, ML: ± 3.3 mm, DV: −4.6 mm) at a speed of 0.03 μl/min. After approximately 3 weeks, optical fibres (core = 200 μm, NA = 0.37) were implanted into the vCA1 (AP: −3.28 mm, ML: ± 3.3 mm, DV: −4.2 mm). After allowing for 7 d to recover, the mice were subjected to behavioural tests. During the social memory test, when the mice sniffed within 2 cm of the inverted cups, 8, 20 or 40 Hz blue light (5 ms pulses, 5–10 mW, 473 nm) was delivered to the vCA1 of vCA1-hTau^CaMKII^ mice or vCA1-hTau^PV^ mice through the optical fibres. The selection of the light stimulation parameters mentioned above is supported by previous reports on excitatory neuron firing during conflict exploratory tasks [30] and motivated behaviours [32], as well as PV neuron firing during social memory tests [33, 34].

### Drug treatment

UA (U6753, Sigma-Aldrich, USA) was dissolved in 90% PBS and 10% Kolliphor (C5135, Sigma-Aldrich, USA). Mice received UA by oral gavage at doses of 15 mg/(kg·d) or 30 mg/(kg·d) for a duration of 1 month.

### Immunostaining

Mice were anaesthetized with 1% sodium pentobarbital and perfused with saline followed by 4% paraformaldehyde. Afterwards, the brains were carefully extracted and fixed in 4% paraformaldehyde overnight. Subsequently, the tissues were transferred to 25% and 30% sucrose solutions for dehydration for 3 d. Coronal sections with a thickness of 30 μm were then obtained using a Leica CM1860 cryostat (Germany).

For immunofluorescence staining, the brain slices were washed with PBS**-**T (PBS containing 0.1% Triton X**-**100) and then incubated with specific antibodies, such as a calcium/calmodulin-dependent protein kinase II (CaMKII) antibody, a PV antibody, or a phosphorylated tau (p-Tau) antibody (pT205 and AT8), at 4 °C for 15–18 h. A transcription factor EB (TFEB) antibody was used in immunostaining experiments of HEK293-hTau cells. Then, the sections or cells were incubated with secondary antibodies at 37 °C for 1 h. Finally, 4′,6-diamidino-2-phenylindole (DAPI) staining was performed. Detailed information regarding the antibodies can be found in Additional file 1: Table S2.

For immunohistochemical 3,3′-diaminobenzidine (DAB) staining, brain slices were pretreated with 3% H_2_O_2_ (in PBS) to eliminate endogenous peroxidase activity. The slices were then dehydrated by a graded ethanol series and sealed with neutral balsam. All images were obtained by a virtual slide microscope (SV120, Olympus, Japan) or a laser scanning confocal microscope (LSM780, Zeiss, Germany). WT, P301L, P301S and 3xTg-AD mice were subjected to AT8 staining. WT and 3xTg-AD mice were subjected to CaMKII and AT8 costaining or PV and pT205 costaining. vCA1-hTau^CaMKII^-ChR2 and vCA1-hTau^PV^-ChR2 mice were subjected to CaMKII staining (*n* = 3 per group).

### Dissection of dCA1 and vCA1 subregions from the hippocampus

We employed a vibratome (Campden 7000 smz, UK) to slice the mouse brains in ice-cold artificial cerebrospinal fluid. Subsequently, for dCA1, brain slices ranging from AP −1.46 mm to AP −2.46 mm were collected, and the hippocampal CA1 region above the midline was manually dissected. Similarly, for vCA1, brain slices spanning from AP −2.92 mm to AP −3.80 mm were collected, and the hippocampal CA1 region below the midline was manually dissected. Finally, the dissected brain tissues were prepared for the relevant experiments. A supplementary graphic depicting the dissection of vCA1 and dCA1 has been included in Additional file 1: Fig. S2. For relevant proteomics and phosphoproteomics experiments, see Additional file 1: Materials and methods.

### Western blotting

Brain tissues (*n* = 3 per group) and cells were isolated and homogenized in ice-cold lysis buffer containing 50 mmol/L Tris–HCl, 100 mmol/L NaCl, 1% Triton X-100, 5 mmol/L ethylene diamine tetraacetic acid, and 1:100 phenylmethylsulfonyl fluoride. The proteins were separated via 10% SDS-PAGE and subsequently transferred onto nitrocellulose membranes (Whatman, UK). After transfer, the membranes were blocked with Western blocking buffer (PBS containing 5% BSA) at room temperature for 30 min. Subsequently, the membranes were incubated with primary antibodies (against HT7, AT8, tau5, β-actin, LC3B, p62 and TFEB) (1:500–1:1000) overnight at 4 °C. The membranes were subsequently incubated with secondary antibodies at room temperature for 1 h. Detailed information regarding the antibodies used is included in Additional file 1: Table S2. Finally, the protein bands on the membranes were visualized using an enhanced chemiluminescence (ECL) system (ChemiScope 6000, Shanghai, China).

### Statistical analysis

The data were analysed and plotted by GraphPad Prism 8. Two-tailed unpaired* t* tests and one-way, two-way or two-way repeated ANOVAs were used, as described in each figure legend. The data are expressed as the mean ± SEM, and *P* < 0.05 was considered to indicate statistical significance.

## Results

### The ventral hippocampus is vulnerable to AD-like tau pathology

**Fig. 1:**
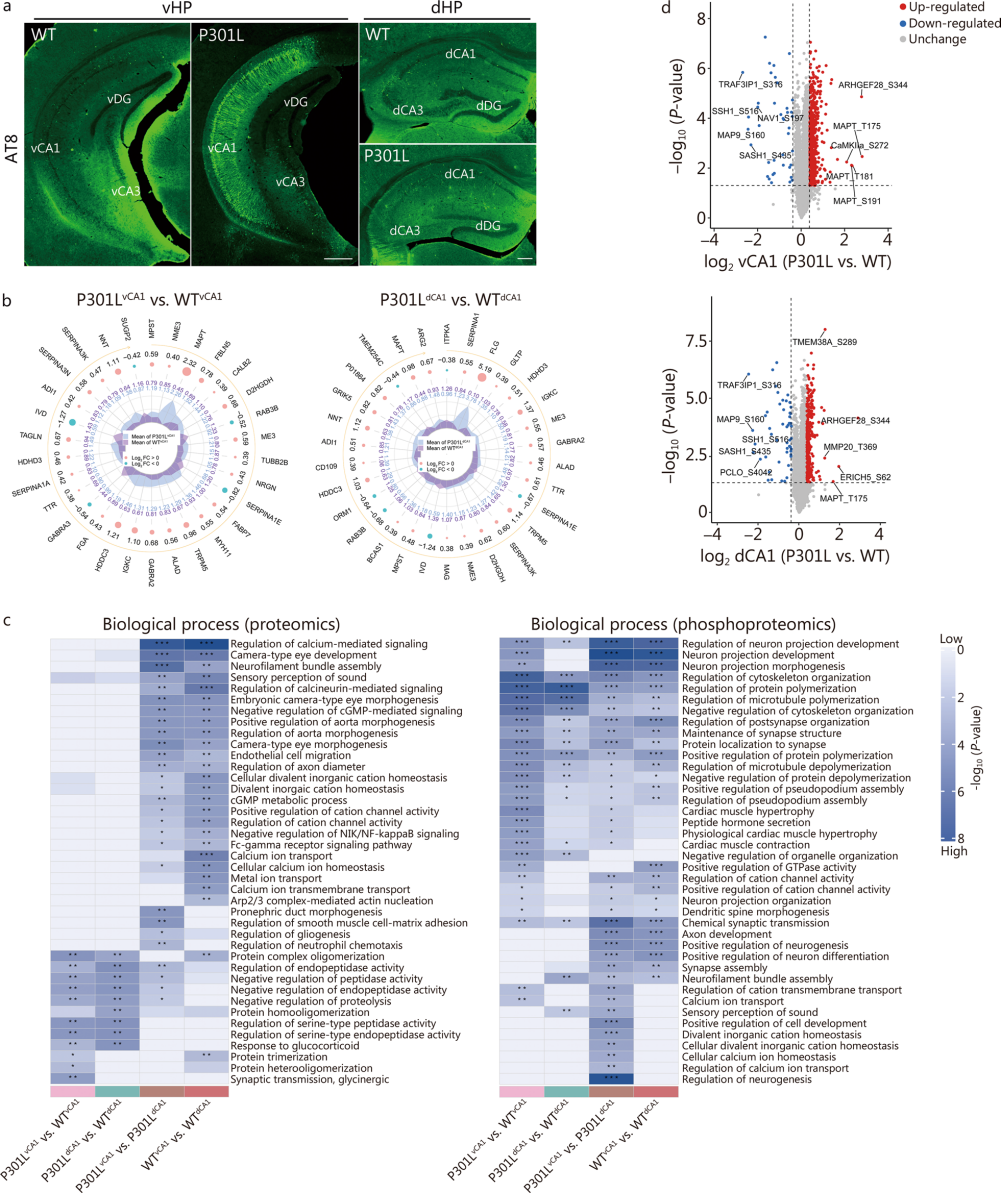
Proteomics and phosphoproteomics of dorsal hippocampal CA1 (dCA1) and ventral hippocampal CA1 (vCA1) in P301L mice. **a** Representative images showing abundant hyper-phosphorylated tau (p-Tau, AT8) in the vCA1 but not in the dCA1 of 3-month-old male P301L mice. Scale bar = 500 μm (vHP) or 200 μm (dHP). **b** Radar graph depicting differentially expressed proteins in the vCA1 (left) or dCA1 (right) between P301L mice and WT mice. From the outside to the inside, the first circle represents the name of protein. The orange arrow in the second circle represents the sequence of the differentially expressed proteins according to their *P*-values, arranged from smallest to largest. The third circle represents the ratio of differential expression changes in the comparison groups, namely, P301L^vCA1^ vs. WT^vCA1^ and P301L^dCA1^ vs. WT^dCA1^, with log_2_ transformation. In this representation, the colour pink indicates upregulation, while blue denotes down-regulation. The size of the data point corresponds to the magnitude of the difference. The fourth circle represents the average quantitative value of the two groups. **c** Heatmap displaying the functional enrichment clusters of differentially expressed proteins and phosphorylated proteins according to biological function. **d** Volcano plot of phosphoproteomic data revealing changes in the levels of phosphorylated proteins in P301L^vCA1^ vs. WT^vCA1^ and P301L^dCA1^ vs. WT^dCA1^. The red and blue dots represent phosphorylated proteins whose abundance significantly increased and decreased, respectively. *n* = 3 per group. ^*^P < 0.05; ^**^P < 0.01; ^***^P < 0.001. WT wild type, vDG ventral dentate gyrus, dDG dorsal dentate gyrus, SERPINA3K serine protease inhibitor A3K, SERPINA3N serine protease inhibitor A3N, ADI1 1,2-dihydroxy-3-keto-5-methylthiopentene dioxygenase, LVD isovaleryl-CoA dehydrogenase, TAGLN transgelin, HDHD3 haloacid dehalogenase-like hydrolase domain-containing protein 3, SERPINA1A serine protease inhibitor A1A, TTR transthyretin, GABRA3 γ-aminobutyric acid receptor subunit alpha-3, FGA fibrinogen alpha chain, HDDC3 guanosine-3’,5’-bis (diphosphate) 3’-pyrophosphohydrolase MESH1, IGKC immunoglobulin kappa constant, GABRA2 γ-aminobutyric acid receptor subunit alpha-2, ALAD delta-aminolevulinic acid dehydratase, TRPM5 transient receptor potential cation channel subfamily M member 5, MYH11 myosin-11, FABP7 fatty acid-binding protein type 7, SERPINA1E serine protease inhibitor A1E, NRGN neurogranin, TUBB2B tubulin beta-2B, ME3 NADP-dependent malic enzyme, RAB3B ras-related protein Rab-3B, D2HGDH D-2-hydroxyglutarate dehydrogenase, CALB2 calretinin, FBLN5 fibulin-5, MAPT microtubule-associated protein tau, NME3 nucleoside diphosphate kinase 3, MPST mercaptopyruvate sulfurtransferase, SUGP2 SURP and G-patch domain-containing protein 2, NNT nicotinamide nucleotide transhydrogenase, TMEM254C transmembrane protein 254c, CD109 CD109 antigen, P01864 Ig gamma-2A chain C region, BCAS1 breast carcinoma-amplified sequence 1 homolog, D2HGDH D-2-hydroxyglutarate dehydrogenase, ITPKA inositol-trisphosphate 3-kinase A, MAP9 microtubule-associated protein 9, SASH1 SAM and SH3 domain-containing protein 1, ARHGEF2 Rho guanine nucleotide exchange factor 2, CAMKIIa calcium/calmodulin-dependent protein kinase type II subunit alpha, TRAF3IP3 TNF receptor-associated factor interacting protein 3, PCLO protein piccolo, TMEM38A trimeric intracellular cation channel type A, ARHGEF28 rho guanine nucleotide exchange factor 28, MMP20 matrix metalloproteinase-20, ERICH5 glutamate-rich protein 5, S serine, T threonine

To outline tau pathology in the hippocampus during AD-like progression, we used an AT8 antibody to detect phosphorylated paired helical filament tau [35] in male 3xTg-AD mice at different ages, specifically focusing on the ventral-dorsal axis (which corresponds to the anterior–posterior axis in humans [36]) of the hippocampus. We found that AT8-immunoreactive (ir) cells were absent in all the hippocampal subregions of nTg mice, even at 12 months of age. In contrast, abundant AT8-ir neurons were already observed in vCA1 but not in dCA1 at 6 months in male 3xTg-AD mice (Additional file 1: Fig. S3). Similarly, in both 3-month-old male and female P301L mice expressing the longest hTau isoform with the FTDP-17 P301L mutation, there was a significant accumulation of hyper-p-Tau (AT8-ir) in cell bodies and neuronal processes of the ventral hippocampus, particularly in vCA1 (Fig. 1a, Additional file 1: Fig. S4a). AT8-ir signals were undetectable in dCA1 (Fig. 1a, Additional file 1: Fig. S4a). The same phenomenon of tau pathology was observed in the hippocampi of 8-month-old male P301S mice (Additional file 1: Fig. S4b), which expresses the P301S mutant form of hTau. These data indicate that vCA1 exhibits greater sensitivity to AD-like tau pathology than dCA1.

To further investigate the differences between vCA1s and dCA1s under physiological conditions and in the presence of AD-like tau pathology, we conducted proteomic and phosphoproteomic analyses in WT and P301L mice. Proteomic analysis revealed 6485 proteins, 158 of which were differentially expressed between the vCA1 and dCA1 in the WT mice (Additional file 1: Fig. S5a). For example, mitochondrial glutamate carrier 2 (SLC25A18), calretinin (CALB2), neuromodulin (GAP43), etc., were up-regulated, while actin-related protein 2/3 complex subunit 5 (ARPC5), neurofilament light polypeptide (NEFL), and Rho GTPase-activating protein 12 (ARHGAP12), among others, were down-regulated in the vCA1 compared with dCA1 in WT mice (Additional file 1: Fig. S5b). These findings indicate the presence of distinct protein networks along the anterior–posterior axis of the hippocampal CA1 region under physiological conditions. As tau accumulated, differential protein expression was observed between vCA1 and dCA1 in P301L mice; this included proteins such as MAPT, secretogranin-2 (SCG2), and fibulin-5 (FBLN5) (Additional file 1: Fig. S5b). Compared with WT mice, there were 50 and 45 differentially expressed proteins in the vCA1 and dCA1 of P301L mice (Additional file 1: Fig. S5a). The top 30 proteins differentially expressed between WT and P301L mice in vCA1 differed from those in dCA1 (Fig. 1b) and included the serine protease inhibitor A3N (SERPINA3N), FBLN5, CALB2, tubulin beta-2B (TUBB2B), neurogranin (NRGN), fatty acid-binding protein (FABP7), myosin-11 (MYH11), fibrinogen alpha chain (FGA), transgelin (TAGLN) and SURP and G-patch domain-containing protein 2 (SUGP2). GO enrichment analyses revealed distinct enriched biological processes, such as protein trimerization/heterooligomerization and synaptic transmission, between WT^vCA1^ and P301L^vCA1^ and between WT^dCA1^ and P301L^dCA1^ (Fig. 1c).

Subsequently, phosphoproteomic analysis was performed to delineate distinct phosphorylated protein networks in the two groups. Compared with dCA1, vCA1 of WT and P301L mice exhibited modification in 644 and 505 proteins and 1021 and 839 sites, respectively (Additional file 1: Fig. S6). The phosphorylation levels of tau at T181 and S191 (tau-T181 and tau-S191) and CaMKIIa at S272 (CaMKIIa-S272) were significantly higher in the vCA1 of P301L mice compared to those in the vCA1 of WT mice (Fig. 1d). However, the phosphorylation level of neuron navigator 1 at S197 (NAV1-S197) in the vCA1 of P301L mice was lower than that in the vCA1 of WT mice (Fig. 1d).

Furthermore, when comparing P301L with WT mice, the differentially phosphorylated proteins in vCA1 were related to the regulation of cytoskeleton organization, the regulation of protein polymerization, the regulation of microtubule polymerization, the negative regulation of cytoskeleton organization, protein localization to synapses, calcium ion transport, etc. (Fig. 1c).

These data delineate distinct protein and phosphorylated protein networks associated with AD-like tau pathology along the anterior–posterior axis of CA1 in the hippocampus.

### Tau^vCA1^ accumulation impairs social memory

Given the association between vCA1 and social behaviours [37, 38], we conducted social exploration and memory tests (Additional file 1: Fig. S7a, b) sequentially on 6-month-old 3xTg-AD mice, which exhibited significant p-Tau accumulation in vCA1. During exploration, both the nTg and 3xTg-AD mice spent more time on the side containing a novel mouse than on the side with an empty cup, demonstrating a preference for social proximity (*P* < 0.01; Additional file 1: Fig. S7c). Notably, nTg mice showed a significant preference for the novel stimulus in the social memory test (*P* < 0.01), but this preference was not observed in 3xTg-AD mice. The 3xTg-AD mice were investigated identically between the familiar mouse and the novel mouse (Additional file 1: Fig. S7d). Moreover, the social discrimination score was significantly lower in 3xTg-AD mice than in nTg mice (*P* < 0.01; Additional file 1: Fig. S7d).

Subsequently, we performed social exploration and memory tests on 3-month-old P301L mice (male) with hyper-p-Tau accumulation in vCA1 but not in dCA1. In social test, both WT and P301L mice displayed a strong preference for social proximity (*P* < 0.01; Additional file 1: Fig. S7e), but P301L mice exhibited identical interaction times between the familiar and novel subjects (*P* > 0.05) with a lower social discrimination score (*P* < 0.01; Additional file 1: Fig. S7f). These findings suggest an association between pathological tau accumulation in vCA1 and social memory deficits in AD mice.

**Fig. 2:**
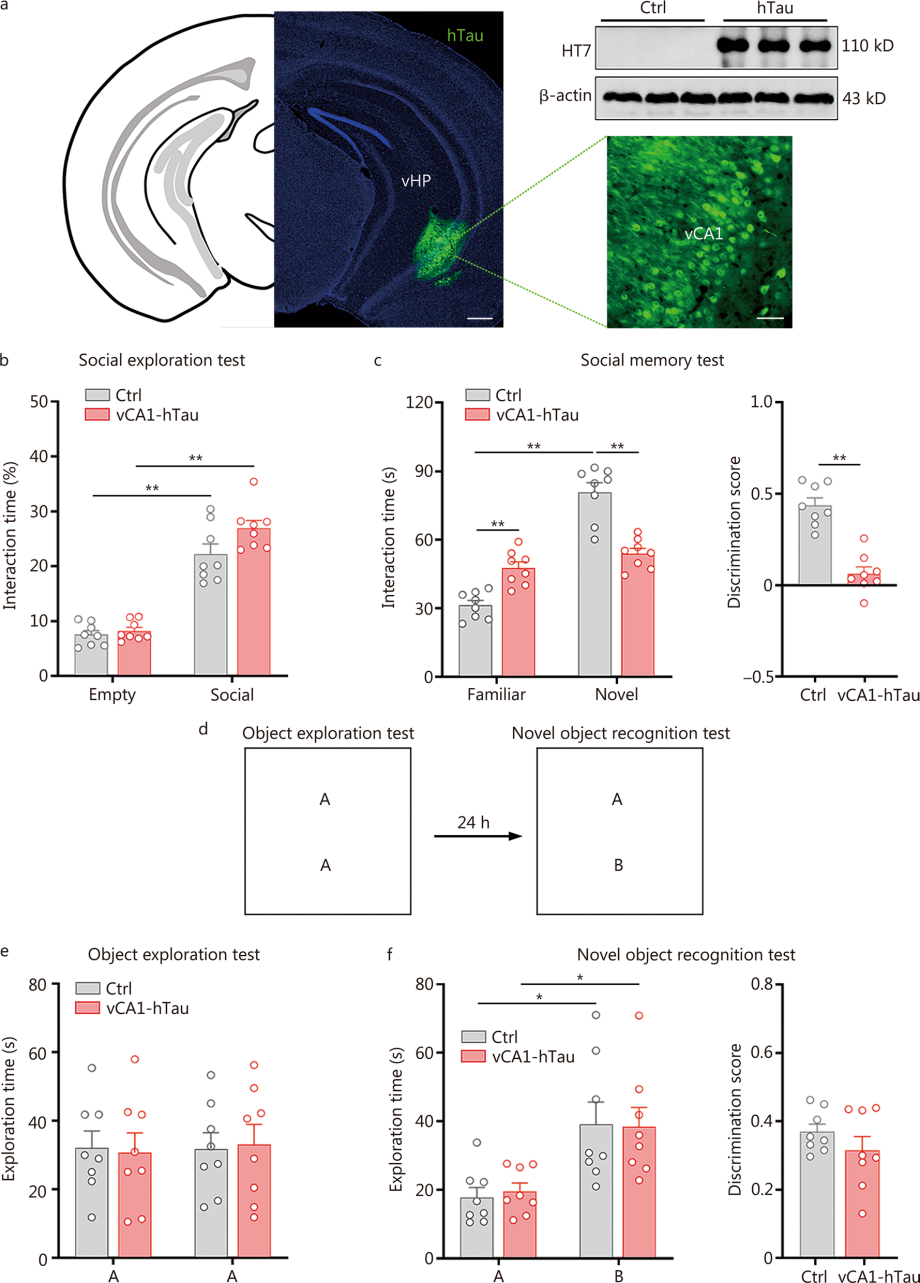
Tau accumulation in vCA1 impairs social memory. **a** Expression of exogenous hTau in vCA1 by the AAV-hSyn-hTau-eGFP virus. Scale bar = 500 μm (vHP) or 50 μm (vCA1). **b** Overexpression of hTau in vCA1 had no effect on sociability. **c** Overexpression of hTau in vCA1 significantly disrupted social preference for novel conspecifics and decreased the discrimination score in social memory test. **d** Behavioural schematic of novel objective recognition tests. **e** There was no difference in the exploration time of object A between the two groups. **f** Both vCA1-hTau and control mice spent more time investigating object B in the test. No difference was detected in the discrimination score between control and vCA1-hTau mice. *n* = 8 per group. ^*^P < 0.05; ^**^P < 0.01, as determined by two-way ANOVA or two-tailed unpaired *t* test. The data are presented as the mean ± SEM. vHP ventral hippocampal, vCA1 ventral hippocampal CA1, hTau human tau

To clarify the causal role of tau accumulation in vCA1 in social memory impairment, we stereotaxically injected AAV-hSyn-hTau-eGFP into vCA1 (vCA1-hTau mice). Four weeks later, the enrichment of hTau in vCA1 was confirmed by immunofluorescence imaging and Western blotting using the hTau-specific antibody HT7 (Fig. 2a, Additional file 1: Fig. S8). Then, we performed social exploration and memory tests. No difference was found between groups in the social exploration test (*P* > 0.05; Fig. 2b). In the social memory test, vCA1-hTau mice spent similar amounts of time exploring familiar and novel conspecifics (*P* > 0.05) and displayed a lower discrimination score than the control group (*P* < 0.01; Fig. 2c). Additionally, there were no significant differences between control and vCA1-hTau groups in object exploration and novel object recognition tests (*P* > 0.05; Fig. 2d–f). In the elevated plus maze test, control and vCA1-hTau mice spent similar amounts of time in the open arm of the elevated plus maze, and the probability of entering into the open arm of the elevated plus maze was the same for both groups (*P* > 0.05; Additional file 1: Fig. S9a). In the open field test, control and vCA1-hTau mice still spent similar amounts of time in the centre zone of the open field, and the travel distances were similar between the two groups (*P* > 0.05; Additional file 1: Fig. S9b). These data indicate that vCA1-hTau mice had no anxiety or complete motor activity.

Furthermore, we overexpressed hTau in the dCA1 of the hippocampus (Additional file 1: Fig. S10a). No significant differences were detected in interaction time or discrimination score (*P* > 0.05; Additional file 1: Fig. S10b, c) between the control group and the dCA1-hTau group, suggesting that dCA1 is not associated with tau-impaired social memory. Taken together, these data suggest that tau accumulation in vCA1 acts as a causal factor that impairs social memory.

### Overexpression of hTau in excitatory neurons impairs recognition of familiar conspecifics

To investigate which type(s) of hippocampal neurons are sensitive to the accumulation of pathological tau and responsible for tau-induced social memory deficits, we conducted double immunostaining on brain slices containing the vCA1 subregion from nTg and 3xTg-AD mice. Compared with those in nTg mice, more AT8 and pT205 signals were colocalized with CaMKII (a marker of excitatory neuron) and PV (a marker of PV neuron), respectively. Quantitative analysis of 6-month-old 3xTg-AD mice revealed that 84% of the AT8^+^ signals were located within CaMKII neurons and 92% of the PV neurons were pT205-ir (Additional file 1: Fig. S11). These data indicate that both excitatory and PV neurons in the vCA1 are susceptible to AD-like tau accumulation.

**Fig. 3:**
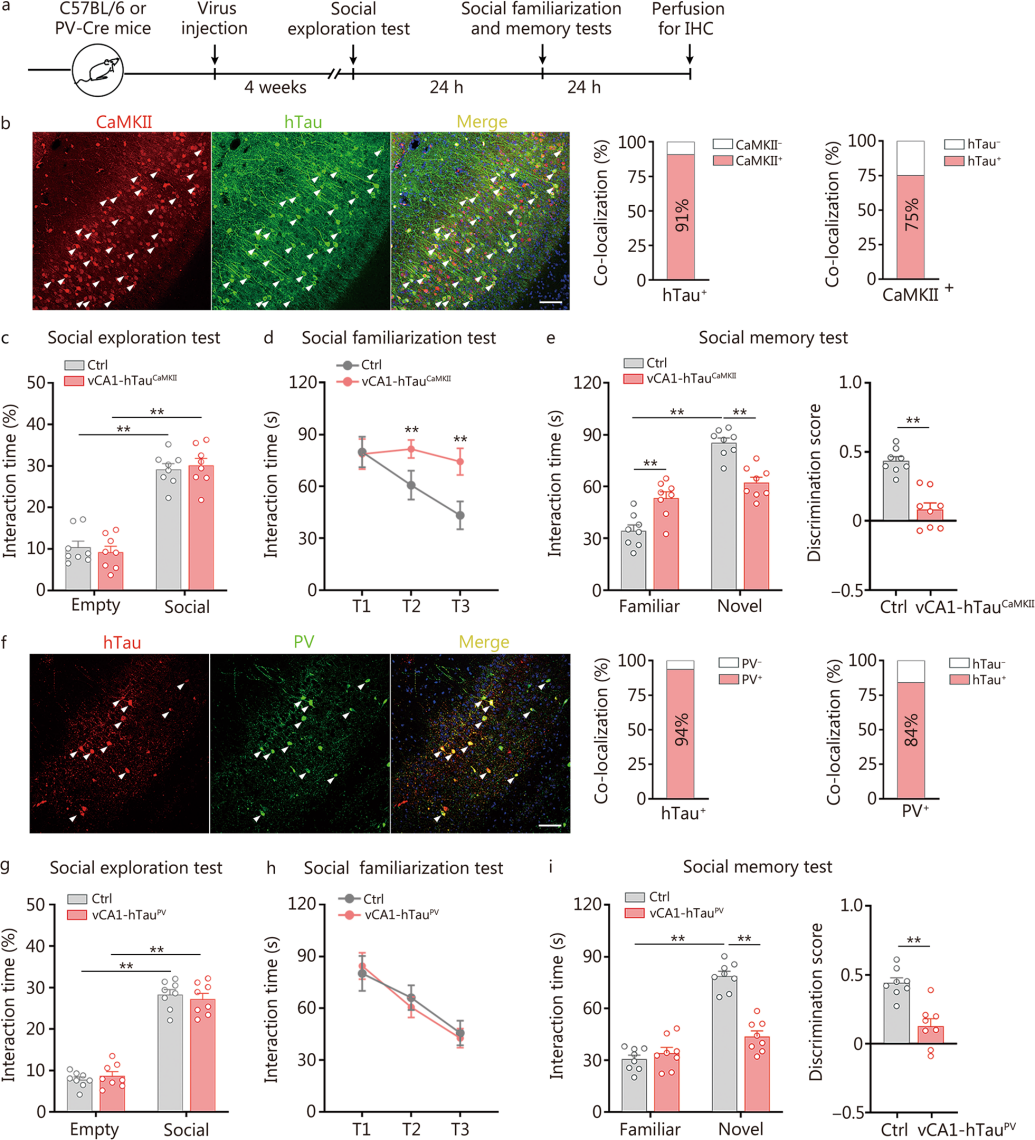
Overexpression of hTau in excitatory and PV neurons impairs social memory. **a** Timeline of the experimental procedures. **b** Representative images showing the expression of AAV-CaMKII-Cre-mCherry and AAV-DIO-hTau-eGFP in the vCA1 of C57BL/6 mice. The arrows indicate colabelled cells. Scale bar = 100 μm. Quantitative analysis revealed that 91% of the hTau^+^ neurons were colocalized with CaMKII and 75% of the CaMKII^+^ neurons were colocalized with hTau. **c** vCA1-hTau^CaMKII^ and control mice spent more time investigating conspecifics than empty cups. **d** vCA1-hTau^CaMKII^ mice failed to familiarize themselves with the same social partner in trials 1 to 3 (T1–T3). **e** In the social memory test, vCA1-hTau^CaMKII^ mice spent similar amounts of time investigating familiar and novel conspecifics. The social discrimination score was lower in the vCA1-hTau^CaMKII^ group. **f** Representative images showing AAV-DIO-hTau-mCherry virus infection (red) and anti-PV immunofluorescence staining (green) in the vCA1 of PV-Cre mice. The arrows indicate colabelled cells. Scale bar = 100 μm. Quantitative analysis revealed that 94% of the neurons with hTau were PV^+^ and 84% of the PV^+^ neurons were colocalized with hTau. **g** Both vCA1-hTau^PV^ group and control group exhibited a preference for conspecifics over empty cups. **h** vCA1-hTau^PV^ mice succeeded in familiarizing themselves with the same social partner in trials 1 to 3 (T1–T3). **i** In the social memory test, vCA1-hTau^PV^ mice spent similar amounts of time investigating familiar and novel conspecifics. The social discrimination score was lower in the vCA1-hTau^PV^ group than in the control group. *n* = 8 per group. ^**^P < 0.01, as determined by two-way ANOVA, two-way repeated ANOVA or two-tailed unpaired *t* test. The data are presented as the mean ± SEM. IHC immunohistochemistry, CaMKII calcium/calmodulin-dependent protein kinase II, PV parvalbumin, vCA1 ventral hippocampal CA1, hTau human tau

To explore whether and how tau accumulation in excitatory neurons disrupts social memory, we infused AAV-CaMKII-Cre-mCherry and AAV-DIO-hTau-eGFP into the vCA1 of C57BL/6 mice (Fig. 3a, b). Four weeks later, 91% of the hTau-expressing neurons were positive for CaMKII, and 75% of the CaMKII neurons exhibited hTau expression (Fig. 3b), indicating the high specificity and efficiency of hTau overexpression in excitatory neurons of the vCA1 via the Cre-LoxP system (referred to as vCA1-hTau^CaMKII^ mice). During social exploration, the vCA1-hTau^CaMKII^ mice performed similarly to the control group (vCA1-mCherry^CaMKII^ mice) (*P* > 0.05; Fig. 3c), indicating complete sociability following hTau overexpression in excitatory neurons of vCA1. Then, prior to the social memory test, we designed and performed familiarization trials in which the mice were continuously exposed to the same social partner during trials 1 to 3. Given the intrinsic preference for novelty, the social interaction time gradually decreased as the familiarization trials continued, indicating familiarization with the same social stimulus. However, no significant difference was detected in familiarization trials of vCA1-hTau^CaMKII^ mice (*P* > 0.05; Fig. 3d), indicating deficits in recognizing familiar subjects. When a novel mouse was introduced to the social memory test, the interaction time between the familiar and novel subjects were the same (*P* > 0.05; Fig. 3e). Compared with controls, the social discrimination score is significantly decreased in vCA1-hTau^CaMKII^ mice (*P* < 0.01; Fig. 3e). These data suggest that hTau accumulation in excitatory neurons of vCA1 may disrupt the encoding, consolidation and/or memory retrieval of social information, thereby impairs social recognition when encountering familiar conspecifics.

### Overexpression of hTau in PV neurons impairs identification of novel conspecifics

To investigate whether and how tau accumulation in PV neurons affects social memory, we stereotaxically infused AAV-DIO-hTau-mCherry into the vCA1 of PV-Cre mice (Fig. 3a, f). Four weeks later, 94% of the hTau-expressing neurons were positive for PV, and 84% of the PV neurons exhibited hTau expression (Fig. 3f), confirming successful overexpression of hTau in PV neurons of the vCA1 via the Cre-LoxP system (referred to as vCA1-hTau^PV^ mice). In behavioural tests, the vCA1-hTau^PV^ mice displayed sociability and social familiarization ability comparable to the control group (*P* > 0.05; Fig. 3g, h). However, social discrimination was markedly impaired in vCA1-hTau^PV^ mice as evidenced by a decrease in interaction time towards novel conspecifics. Meanwhile, the discrimination score was robustly decreased in vCA1-hTau^PV^ mice compared with controls (*P* < 0.01; Fig. 3i). Collectively, these data demonstrate that tau accumulation specifically in the PV neurons of vCA1 impairs the identification of novel conspecifics without affecting familiar recognition.

### Tau accumulation impairs discrimination-associated firing of excitatory and PV neurons in the vCA1

**Fig. 4:**
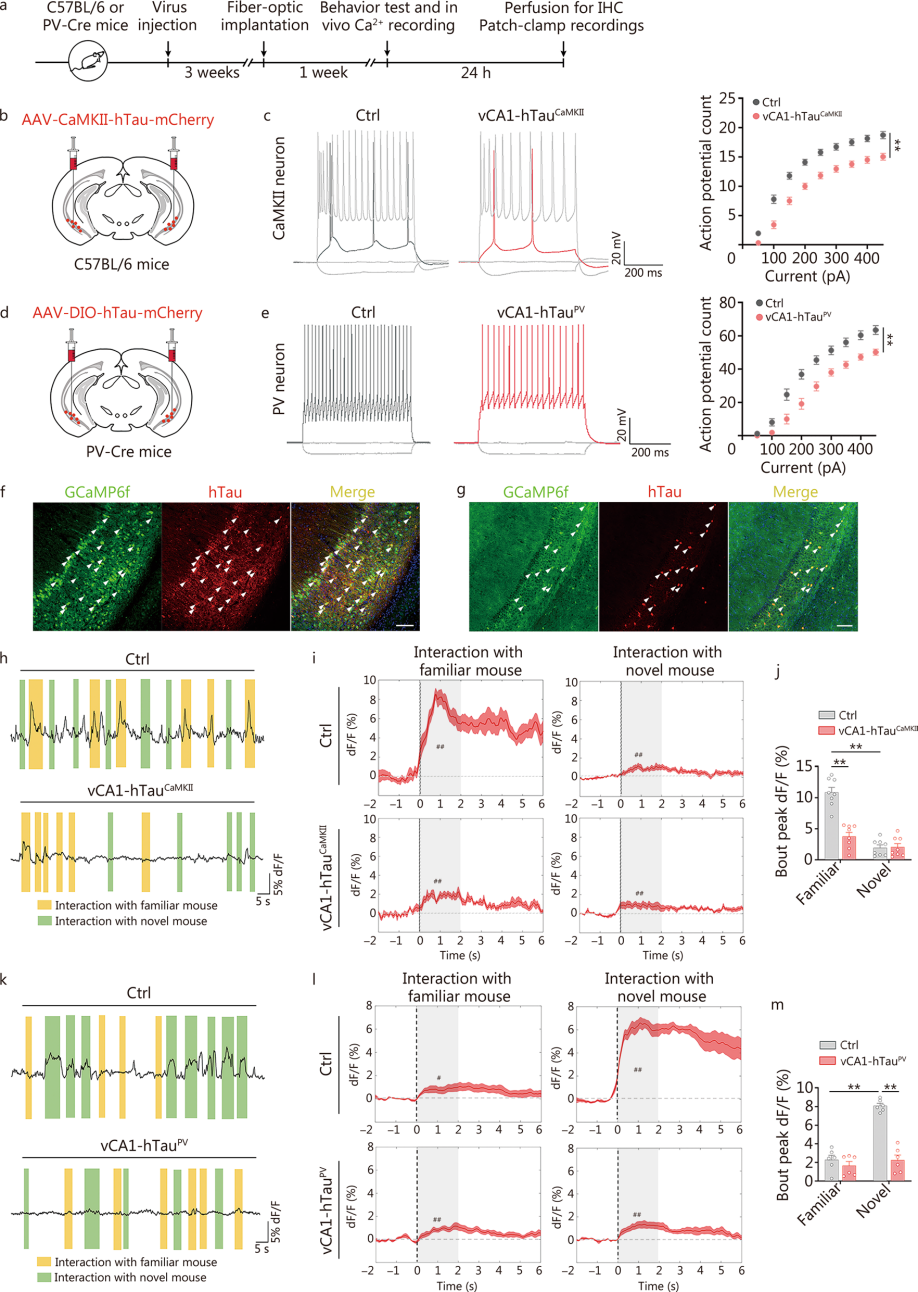
Tau accumulation disrupts the discharge of excitatory and PV neurons. **a** Timeline of the experimental procedures. **b** Schematic of AAV-CaMKII-hTau-mCherry injection into the vCA1 of C57BL/6 mice. **c** Representative traces of action potentials of CaMKII^+^ neurons from the control and vCA1-hTau^CaMKII^ groups following the injection of currents. Ctrl: *n* = 19 neurons from 4 mice; vCA1-hTau^CaMKII^: *n* = 22 neurons from 5 mice. **d** Schematic of AAV-DIO-hTau-mCherry injection into the vCA1 of PV-Cre mice. **e** Representative traces of action potentials of PV neurons from the control and vCA1-hTau^PV^ groups following the injection of currents. Ctrl: 21 neurons from 5 mice; vCA1-hTau^PV^: 24 neurons from 5 mice. Representative image of GCaMP6f expression in CaMKII-hTau^+^ (**f**) and PV-hTau^+^ (**g**) neurons in the vCA1. The arrows indicate colabelled cells. Scale bar = 100 μm. **h** Representative traces of GCaMP6f signals in the control and vCA1-hTau^CaMKII^ groups. **i** Per-bout stacked plots of CaMKII-GCaMP6f signals were aligned to the start of the interaction event. The presence of “#” indicates a significant increase in the average calcium signals during the social exploration period (the first 2 s) compared to the baseline period (2 s before social exploration).* n* = 8 per group. **j** Quantitative analysis of peak dF/F of Ca^2+^ signals in CaMKII^+^ neurons during the social memory test. **k** Representative traces of GCaMP6f signals in the control and vCA1-hTau^PV^ groups. **l** Per-bout stacked plots of PV-GCaMP6f signals aligned to the start of the interaction event. The presence of “#” indicates a significant increase in the average calcium signals during the social exploration period (the first 2 s) compared to those during the baseline period (2 s before social exploration). *n* = 6 per group. **m** Quantitative analysis of peak dF/F of Ca^2+^ signals in PV^+^ neurons during the social memory test.* n* = 6 mice per group. ^*^P < 0.05; ^**^P < 0.01, as determined by two-way ANOVA, two-way repeated ANOVA, two-tailed unpaired *t* test or two-tailed *t* test. The data are presented as the mean ± SEM. IHC immunohistochemistry, CaMKII calcium/calmodulin-dependent protein kinase II, PV parvalbumin, vCA1 ventral hippocampal CA1, GCaMP6f genetically encoded calcium indicators, hTau human tau

To investigate how accumulated tau affects the activities of excitatory and PV neurons in the vCA1, we first performed in vitro patch-clamp recordings to assess the changes in excitability after hTau overexpression (Fig. 4a). In response to step-current injection, both excitatory (*P* < 0.01; Fig. 4b, c) and PV neurons (*P* < 0.01; Fig. 4d, e) exhibited a significant decrease in intrinsic excitability. The threshold of action potential was elevated after hTau overexpression (*P* < 0.05 or *P* < 0.01; Additional file 1: Fig. S12a, c). However, the capacitance membrane (Cm) and resting membrane potential (RMP) did not significantly change between the two groups (*P* > 0.05; Additional file 1: Fig. S12b, d). These data indicate hypoactivity of excitatory and PV neurons in response to intracellular hTau accumulation. No hTau signals were detected in PV neurons of vCA1-hTau^CaMKII^ mice (Additional file 1: Fig. S13). Moreover, there were no significant changes in the neuronal activities or passive properties of excitatory neurons (CaMKII^+^) in vCA1-hTau^PV^ mice (*P* > 0.05; Additional file 1: Fig. S14a-c) or inhibitory neurons (PV^+^) in vCA1-hTau^CaMKII^ mice (*P* > 0.05; Additional file 1: Fig. S14d-f), suggesting hTau is rarely released or transmitted following its overexpression.

Next, we analysed the real-time activity of excitatory and PV neurons in the vCA1 during social discrimination by in vivo calcium recording (Fig. 4f-m). Using the CaMKII promoter, GCaMP6f and hTau were coexpressed in excitatory neurons (Fig. 4f, Additional file 1: Fig. S15a). Employing a Cre-dependent strategy, GCaMP6f and hTau were targeted to PV neurons in vCA1 (Fig. 4g, Additional file 1: Fig. S15b). The Ca^2+^ fluorescence signals were continuously recorded in the arena after the introduction of novel and familiar mice. For excitatory neurons in vCA1, we observed a robust increase in Ca^2+^ signals when control mice explored a familiar partner (Fig. 4h, i). However, these familiar conspecific recognition-associated discharges were markedly inhibited in vCA1-hTau^CaMKII^ mice (Fig. 4h, i). Quantitative analysis revealed that in the vCA1-hTau^CaMKII^ group, the peak Ca^2+^ signals during interaction with familiar mice decreased to 34% of the control level (*P* < 0.01; Fig. 4j). There was no significant reduction in the number of discharges associated with novel conspecific recognition in vCA1-hTau^CaMKII^ mice (Fig. 4h-j). Tau accumulation had no effect on eGFP signals in excitatory neurons in the vCA1 (Additional file 1: Fig. S16a). These findings demonstrate that tau accumulation in vCA1 suppresses the discharge activity of excitatory neurons specifically during familiar conspecific recognition.

Interestingly, PV neurons in vCA1 displayed a different discharge pattern during social discrimination. Prominent Ca^2+^ transients were observed in the PV neurons of control mice during the exploration of a novel mouse, but these transients were significantly suppressed in vCA1-hTau^PV^ mice (Fig. 4k, l). The peak Ca^2+^ signals during interaction between vCA1-hTau^PV^ mice with novel mice decreased to 22% of the control group (*P* < 0.01; Fig. 4m), indicating insufficient activation of vCA1-hTau^PV^ neurons during novel identification. However, no significant difference was observed between control and vCA1-hTau^PV^ mice when investigating familiar mice (Fig. 4k-m). Tau accumulation also did not affect eGFP signals in PV neurons (Additional file 1: Fig. S16b).

**Fig. 5:**
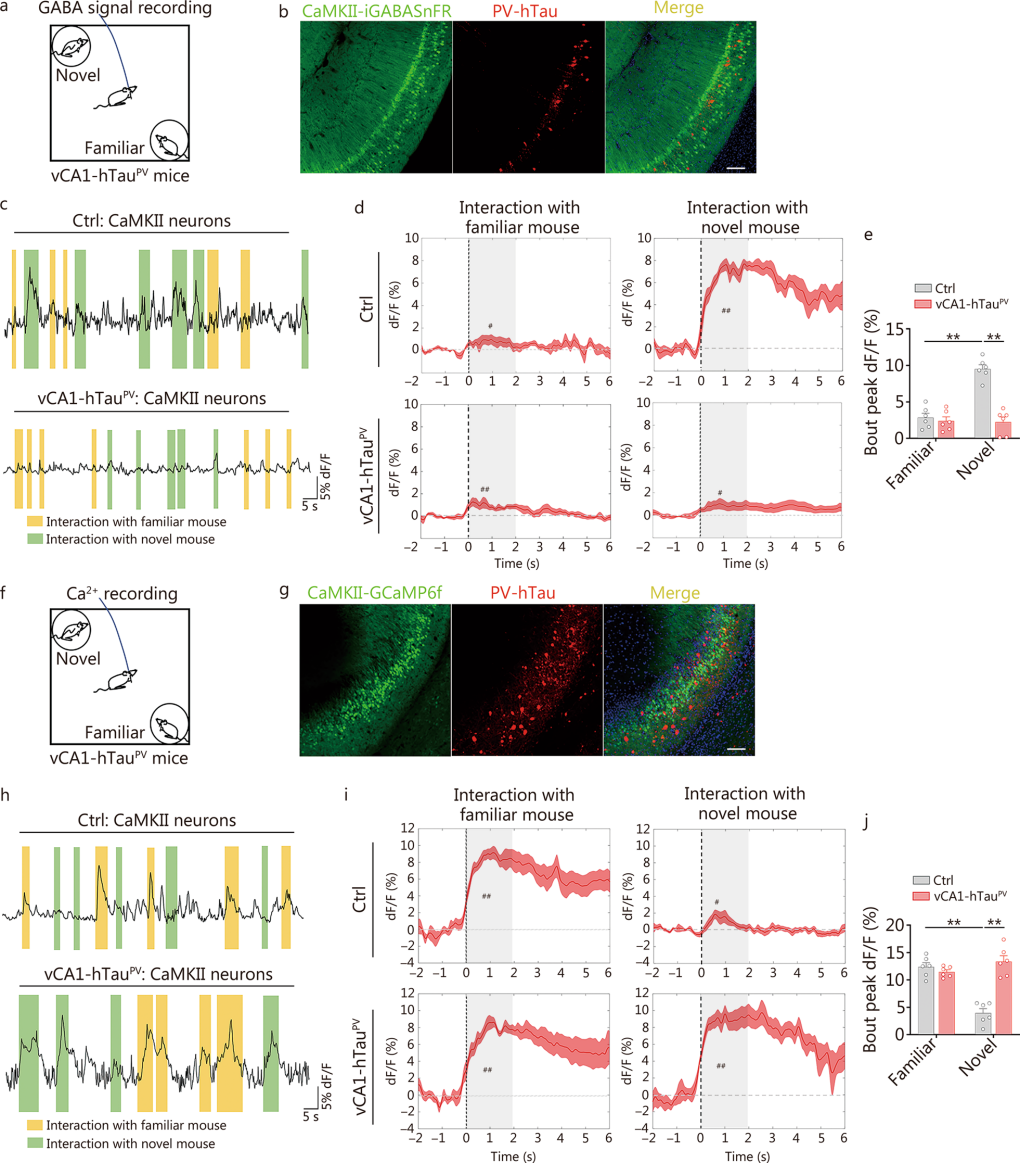
vCA1-hTau^PV^ accumulation disinhibits excitatory neurons during novel conspecific identification. **a** Schematic of GABA signal recording in the social memory test. **b** Representative image of iGABASnFR and hTau expression in the vCA1 of PV-Cre mice. Scale bar = 100 μm. **c** Representative trace of GABA signals. **d** Per-bout stacked plots of GABA signals on CaMKII^+^ neurons aligned to the start of the interaction event. The presence of “#” indicates a significant increase in the average GABA signals during the social exploration period (the first 2 s) compared to those during the baseline period (2 s before social exploration). **e** Quantitative analysis of peak dF/F of GABA signals into CaMKII^+^ neurons during social memory test. **f** Schematic of Ca^2+^ signal recording in the social memory test. **g** Representative image of GCaMP6f and hTau expression in the vCA1 of PV-Cre mice. Scale bar = 100 μm. **h** Representative trace of GCaMP6f signals. **i** Per-bout stacked plots of GCaMP6f signals of CaMKII^+^ neurons aligned to the start of the interaction event. The presence of “#” indicates a significant increase in the average calcium signals during the social exploration period (the first 2 s) compared to those during the baseline period (2 s before social exploration). **j** Quantitative analysis of peak dF/F of Ca^2+^ signals in CaMKII^+^ neurons during the social memory test. *n* = 6 per group. ^*^P < 0.05; ^**^P < 0.01 as determined by two-way ANOVA or two-tailed paired *t* test. The data are presented as the mean ± SEM. CaMKII calcium/calmodulin-dependent protein kinase II, PV parvalbumin, vCA1 ventral hippocampal CA1, iGABASnFR intensity-based GABA-sensing fluorescence reporters, GCaMP6f genetically encoded calcium indicators

Given the inhibitory effects of PV neurons on local excitatory neurons via the neurotransmitter GABA [39–41], we further recorded fluorescent GABA neurotransmitter sensors in vivo to investigate whether GABA release into excitatory neurons changes under the accumulation of vCA1-hTau^PV^, and how this change contributes to deficits in social memory caused by vCA1-hTau^PV^. AAV-CaMKII-iGABASnFR, a genetically engineered GABA sensor, was used to target excitatory neurons invCA1, and the real-time GABA signals of vCA1-hTau^PV^ mice were monitored during the whole social memory test (Fig. 5a, b). In the control group, the fluorescence intensity of iGABASnFR was significantly greater when the mice explored novel mice than when they explored familiar mice (Fig. 5c, d). However, this novel identification-associated GABA release was markedly suppressed in the vCA1-hTau^PV^ group, and there was a significant reduction in the peak GABA signals when the vCA1-hTau^PV^ mice were exposed a novel mouse (*P* < 0.01; Fig. 5e). Quantitative analysis revealed that the area under the ΔF/F curves of vCA1-CaMKII-GABA signals (GABA signals on excitatory neurons of vCA1) during interaction with a novel mouse in the vCA1-hTau^PV^ mouse group was significantly decreased compared with the control group (*P* < 0.01; Additional file 1: Fig. S17a). Furthermore, we injected AAV-CaMKII-GCaMP6f into vCA1 and conducted population calcium recording to examine the real-time activity of vCA1 excitatory neurons in the presence of insufficient GABA release from PV neurons of vCA1-hTau^PV^ mice (Fig. 5f, g). No significant changes were observed during the interaction with a familiar conspecific, while the average Ca^2+^ signals increased robustly when the vCA1-hTau^PV^ mice explored a novel mouse compared with the control group (Fig. 5h, i). Quantitative analysis revealed that the peak Ca^2+^ signals and area under the ΔF/F curves of CaMKII^+^ neurons in the vCA1 during interaction with a novel mouse were markedly greater in the vCA1-hTau^PV^ group than in the control group (*P* < 0.01; Fig. 5j, Additional file 1: Fig. S17b). No significant fluctuations were detected in the eGFP signals (Additional file 1: Fig. S18). These data indicate that hTau accumulation in PV neurons hyperactivates local excitatory neurons via disinhibition during novel conspecific identification.

Collectively, these data demonstrate that hTau accumulation inhibits and disrupts the discharge of excitatory and PV neurons in vCA1. The accumulation of hTau in excitatory neurons impairs social memory by suppressing familiar conspecific recognition-associated discharge of excitatory neurons, whereas hTau accumulation in PV neurons leads to the misidentification of a novel identical familiar feature by non-inhibition in excitatory neurons of the vCA1.

### Photoactivation of excitatory and PV neurons in the vCA1 rescues hTau accumulation-impaired social memory

**Fig. 6:**
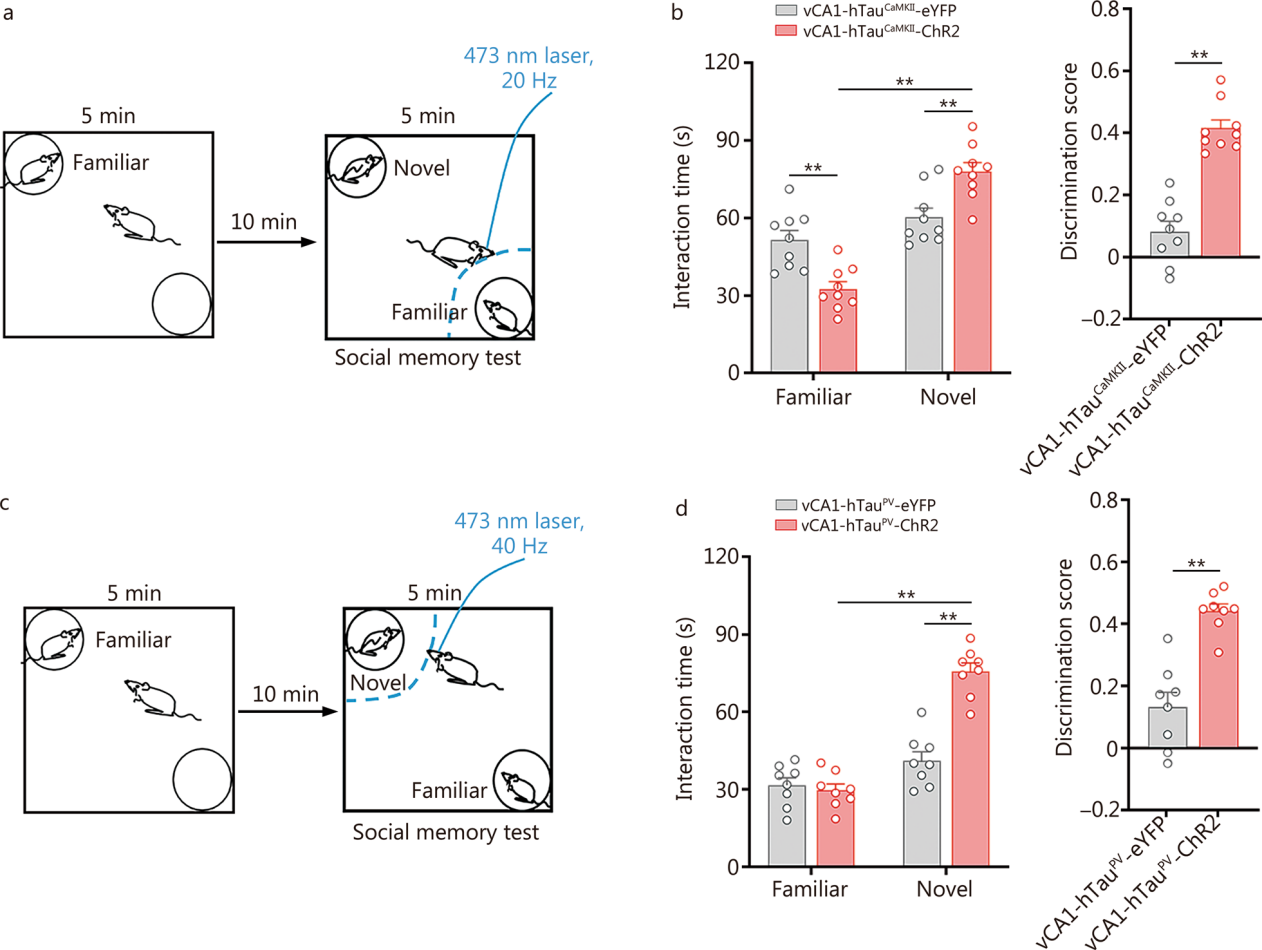
Photoactivation of excitatory and PV neurons rescues tau accumulation-impaired discrimination in social memory. Schematic of stimulation while vCA1-hTau mice were investigating familiar (**a**) and novel (**c**) conspecifics in the social memory test. **b** When vCA1-hTau^CaMKII^ mice were exploring a familiar mouse, photoactivation of excitatory neurons in the vCA1 significantly increased the preference for the novel mouse and concurrently raised the discrimination score. *n* = 9 per group. **d** Photostimulation of PV neurons in the vCA1 at 40 Hz, as in which vCA1-hTau^PV^ mice were exposed a novel mouse, efficiently increased the social interaction time with the novel mouse and increased the discrimination score. *n* = 8 per group. ^**^P < 0.01, as determined by two-way ANOVA or two-tailed unpaired *t* test. The data are expressed as the mean ± SEM. CaMKII calcium/calmodulin-dependent protein kinase II, PV parvalbumin, vCA1 ventral hippocampal CA1, eYFP enhanced yellow fluorescent protein, ChR2 channelrhodopsins-2

Next, we explored whether optogenetic manipulation of excitatory and PV neurons in vCA1 could ameliorate social memory loss induced by vCA1-hTau accumulation. We injected AAV-CaMKII- hChR2(H134R)-eYFP and AAV-EF1a-DIO-hChR2(H134R)-eYFP into the vCA1 to target excitatory and PV neurons, respectively. Ninety-seven percent of excitatory and 94% of PV neurons in vCA1 were ChR2 positive (Additional file 1: Fig. S19), suggesting that ChR2 targets the vast majority of these two types of neurons in vCA1. The photoreactivity of vCA1-CaMKII-ChR2 neurons was verified by ex vivo brain slice recording (Additional file 1: Fig. S20a). During the social memory test, vCA1-hTau^CaMKII^-ChR2 mice were subjected to 20 Hz light stimulation to activate the vCA1 excitatory neurons as they approached a familiar partner (Fig. 6a) and robustly increased interaction time with a novel conspecific (*P* < 0.01; Fig. 6b). The vCA1-hTau^CaMKII^-ChR2 group exhibited a higher discrimination score than the vCA1-hTau^CaMKII^-eYFP group upon light stimulation (*P* < 0.01; Fig. 6b). However, photostimulating excitatory neurons in the vCA1 during the familiarization stage did not show any improvements (*P* > 0.05; Additional file 1: Fig. S20b, c). In another control group of mice expressing eYFP only, no beneficial effect was observed in hTau^CaMKII^ mice when the light was on (*P* > 0.05; Additional file 1: Fig. S21a). These data indicate that targeting excitatory neurons during the retrieval phase of social memory, rather than the encoding phase of social information, is the critical time window for rescuing social memory impairment induced by vCA1-hTau^CaMKII^ accumulation.

Next, we photoactivated PV neurons in the vCA1 at 40 Hz when vCA1-hTau^PV^ mice were approaching a novel mouse (Fig. 6c). A significant increase in interaction time with a novel conspecific and discrimination score was detected in the vCA1-hTau^PV^-ChR2 group (*P* < 0.01; Fig. 6d). In another control group of mice expressing eYFP only, no beneficial effects were observed in vCA1-hTau^PV^ mice when the light was on (*P* > 0.05; Additional file 1: Fig. S21b). These data suggest that specifically activating PV neurons during the recognition of novel conspecifics can effectively correct the improper activation of excitatory neurons in the presence of vCA1-hTau^PV^ and rescue the social memory impairment caused by vCA1-hTau^PV^ accumulation.

No further improvements in social discrimination were found when we photostimulated excitatory or PV neurons from the control group at different rhythms (*P* > 0.05; Additional file 1: Figs. S22, S23), suggesting ceiling effects under physiological conditions.

### UA reduces pathological tau load and rescues tau-impaired social memory

**Fig. 7:**
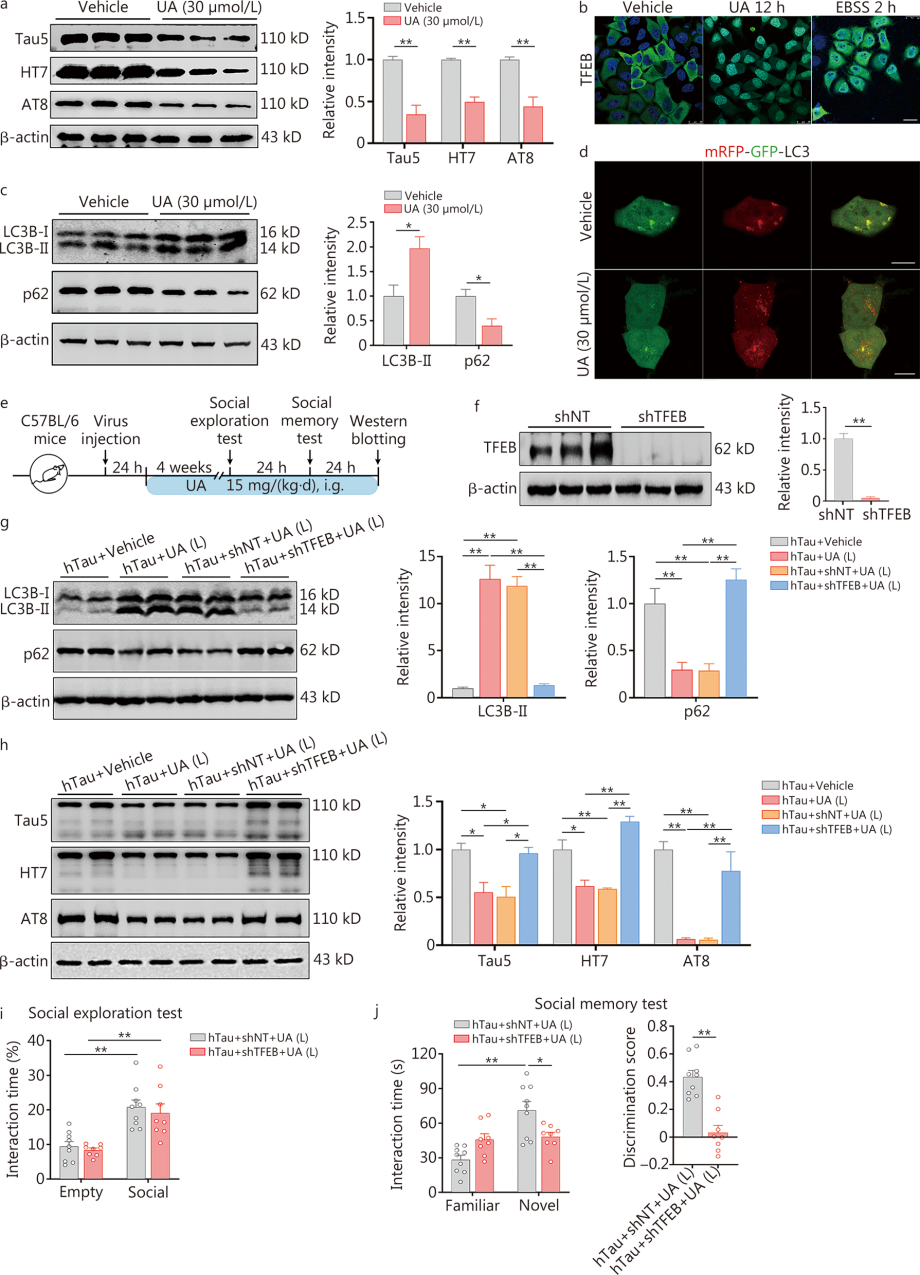
Ursolic acid (UA) reduces the pathological tau load to improve social memory via TFEB. **a** Treatment with 30 μmol/L UA markedly reduced the total tau (tau5, HT7) and phosphorylated tau (p-Tau, AT8) levels in primary neurons instantly transferred with hTau, determined by Western blotting. **b** Treatment with 30 μmol/L UA for 12 h increased the translocation of TFEB from the cytoplasm to the nucleus, as detected by immunostaining. Green: TFEB; blue: DAPI. Scale bar = 25 μm. **c** UA treatment significantly increased LC3B-II and decreased p62 levels in HEK293-hTau cells, as determined by Western blotting. **d** Increased colocalization of mRFP and GFP was observed in HEK293-hTau cells transfected with the tandem fluorescent mRFP-GFP-LC3 (tfLC3) construct, indicating that an increased number of autophagosomes did not fuse with the lysosome. The number of red puncta (mature autolysosomes) increased in the UA-treated group, suggesting that UA promoted autophagy. Scale bar = 10 μm. **e** Timeline of the experimental procedures. **f** Knocking down *TFEB* effectively decreased TFEB levels in vCA1-hTau mice. **g**, **h** Knocking down *TFEB* attenuated UA-induced increases in LC3B-II, decreases in p62, total tau (tau5, HT7) and phosphorylated tau (p-Tau, AT8) in vCA1-hTau mice. **i** Knocking down *TFEB* had no significant effect on sociability. **j** Knocking down *TFEB* effectively attenuated the UA-induced increase in interaction time with novel mice and social discrimination score in vCA1-hTau mice. hTau + shNT + UA (L): *n* = 9; hTau + shTFEB + UA (L): *n* = 8. ^*^P < 0.05; ^**^P < 0.01, as determined by one-way ANOVA, two-way ANOVA or two-tailed unpaired *t* test. The data are expressed as the mean ± SEM. L low-dose, TFEB transcription factor EB, mRFP mCherry red fluorescence protein, GFP green fluorescence protein, LC3B microtubule-associated proteins 1A/1B light chain 3B, p62 sequestosome-1, i.g. intragastric administration

Next, we aimed to identify compound(s) that efficiently alleviate tau toxicity in social memory. We discovered that UA, a triterpenoid compound with a molecular weight of 456.7 kD that is naturally found in fruit peels, herbs, and spices, exhibited profound decreases in total tau (tau5, HT7) and p-Tau (AT8) levels at multiple sites associated with AD in primary neurons instantly transfected with hTau and HEK293 cells with stable expression of WT full-length hTau (termed HEK293-hTau) (*P* < 0.05; Fig. 7a, Additional file 1: Fig. S24a). No significant reduction in *tau* mRNA was observed in the presence of UA at 30 μmol/L (*P* > 0.05; Additional file 1: Fig. S24b). Moreover, we did not observe changes in cell viability in HEK293-hTau cells after UA treatment until 100 μmol/L (*P* < 0.05; Additional file 1: Fig. S25). These data demonstrated that UA, at a relatively safe dosage, can effectively reduce both total tau and hyper-p-Tau levels in vitro.

Autophagy is a conserved mechanism that cells utilize to degrade long-lived proteins and organelles through lysosome-mediated degradation. TFEB, a master regulator of autophagy and the lysosomal pathway, has recently been reported to selectively clear aberrant tau proteins [42]. To explore the involvement of TFEB, we employed HeLa cells stably expressing the 3x-Flag-TFEB construct to examine the effects of UA on TFEB translocation. Compared with the vehicle group, UA treatment at 30 μmol/L for 12 h markedly increased the translocation of TFEB from the cytoplasm to the nucleus, suggesting TFEB activation by UA (Fig. 7b). Moreover, UA treatment significantly enhanced autophagy in HEK293-hTau cells, as indicated by increased LC3B-II and decreased p62 levels (*P* < 0.05; Fig. 7c). To monitor autophagic flux per se, a tandem labeled mRFP-GFP-LC3 (tfLC3) reporter was used to transfect in HEK293-hTau cells, we observed that the number of autophagosomes (yellow-fluorescent puncta) were increased in vehicle group (Fig. 7d). However, the red-fluorescent puncta were predominant after UA treatment at 30 μmol/L (Fig. 7d), suggesting increased autophagolysosome formation. Taken together, these data demonstrated that UA could activate TFEB and simultaneously enhance autophagy by promoting autophagolysosome formation.

Then, we tested the effects of UA on autophagy promotion and tau reduction in vivo. vCA1-hTau mice received either low-dose UA [15 mg/(kg·d)] or high-dose UA [30 mg/(kg·d)] by oral gavage for 28 d. Compared with the high-dose group [hTau + UA (H)], the low-dose UA treatment markedly reduced the total tau (tau5), hTau (HT7) and p-Tau (AT8) protein levels (*P* < 0.05 or *P* < 0.01; Additional file 1: Fig. S26a). According to the results of social exploration and memory tests, low-dose UA treatment markedly ameliorated social memory deficits in vCA1-hTau mice, as evidenced by increased social exploration towards a novel mouse and a higher discrimination score (*P* < 0.01; Additional file 1: Fig. S26b, c). No toxic effect on social memory were detected in C57BL/6 mice after low-dose UA treatment (*P* > 0.05; Additional file 1: Fig. S27).

To investigate whether TFEB mediates the beneficial effects of UA on tau reduction, autophagy enhancement and memory improvement in vivo, we injected AAV-hSyn-shTFEB into vCA1 to knock down *TFEB* (*P* < 0.01; Fig. 7e, f). Autophagy promotion (*P* < 0.01; Fig. 7g), a decrease in tau load (*P* < 0.05; Fig. 7h), cell excitability recovery (*P* < 0.01; Additional file 1: Fig. S28), and improvements in social memory (*P* < 0.01; Fig. 7i, j) of UA were abolished in vCA1-hTau mice after *TFEB* knockdown.

Taken together, these results demonstrated that UA treatment promoted autophagy to reduce aberrant tau load and fundamentally rescued tau accumulation-impaired social memory in a TFEB-dependent manner.

## Discussion

**Fig. 8:**
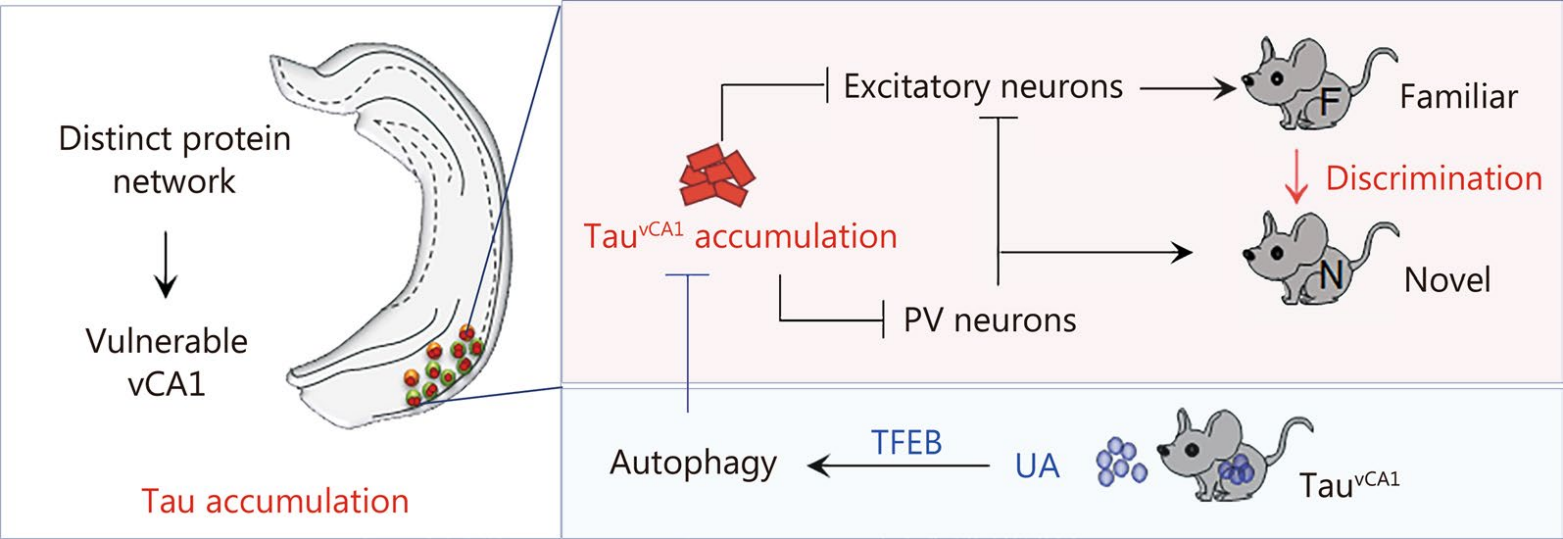
Proposed working model of how to target vulnerable microcircuits in the ventral hippocampus to ameliorate Alzheimer-like social memory loss. Proteomic and phosphoproteomic analyses revealed the characteristics of ventral hippocampal CA1 (vCA1) under both physiological conditions and in the presence of AD-like tau pathology. Excitatory and PV neurons in the vCA1 are vulnerable to tau accumulation. Accumulated tau suppresses excitability and disrupts the firing patterns of excitatory and PV neurons, impairing social memory. Intragastric administration of UA efficiently promoted autophagy to reduce the accumulation of tau and improve social memory in a TFEB-dependent manner. vCA1 ventral hippocampal CA1, PV parvalbumin, TFEB transcription factor EB, UA ursolic acid, AD alzheimer’s disease

To our knowledge, this was the first study to identify the association of distinct protein and phosphorylated protein networks with vulnerable vCA1 in response to AD-like tau pathology. Tau proteins are more likely to mislocate and accumulate in the cell body and dendrites of neurons in the vCA1, particularly in excitatory and PV neurons. Both general and neuron-specific (for excitatory and PV neurons, respectively) overexpression of hTau in vCA1 significantly impaired social memory. Intriguingly, overexpressing hTau in excitatory and PV neurons of vCA1 suppressed familiar conspecific recognition-associated and novel conspecific identification-associated firings, respectively. Furthermore, hTau accumulation in PV neurons disinhibited local excitatory neurons via insufficient GABA release specifically during novel conspecific identification. Photostimulation of excitatory and PV neurons efficiently ameliorated tau-induced impairments in social memory. Interestingly, UA, a triterpenoid compound, markedly reduced the accumulation of tau, recovered neural excitability within the vCA1 microcircuit and ameliorated AD-like social memory deficits by promoting autophagy in a TFEB-dependent manner. Therefore, the present study not only highlights the vulnerability of the ventral hippocampus and its distinct neurons to tau accumulation but also uncovers a microcircuit mechanism underlying tau-impaired social memory. Furthermore, the novel findings regarding the efficiency of UA and the pivotal role of TFEB in tau reduction may provide a promising strategy for preventing AD in the future (Fig. 8).

Tau protein predominantly aggregates in the brains of patients with AD [43, 44]. By positron emission computed tomography (PET) and magnetic resonance imaging (MR), the hippocampus was found to be one of the most vulnerable brain regions to tau pathologies [12, 45, 46]. Intriguingly, within the human hippocampus, tau appears to accumulate in the anterior region before the posterior region of the hippocampus [12]. Many previous studies have focused on dysfunction of the dorsal hippocampus in AD. Here, we unexpectedly found that the ventral hippocampus, especially its vCA1 subset, had a greater accumulation of mislocated p-Tau than the dorsal hippocampus. Proteomic analysis revealed more SERPINA3N in vCA1 of P301L mice than WT mice. SERPINA3N is a serine protease inhibitor whose expression is significantly greater in the NFTs of AD brains than in those of control brains [47]. Injecting SERPINA3N into the hippocampal CA1 region of P301S mice induced oligomerization of tau proteins without altering their monomers [47]. Therefore, we speculate that increased SERPINA3N may be associated with tau pathology in the vulnerable vCA1 of P301L mice. By phosphoproteomics, hyperphosphorylated CaMKIIa at Ser272 and hypo-phosphorylated NaV1 at Ser197 were detected in vCA1 of P301L mice compared with WT mice. Considering the association of CaMKIIa with NFT in AD [48, 49] and the facilitating effects of NaV1 on microtubule-actin cross talk [50], it is plausible that CaMKIIa and NaV1 may play a role in AD-like tau pathology within the vCA1, either independently or in combination. These potential mechanisms warrant further investigation to fully understand the vulnerability of vCA1 to AD-like tau pathology.

We further revealed that both excitatory and PV neurons in vCA1 were vulnerable to the accumulation of p-Tau. These findings were in line with previous reports on interneurons in the hippocampal dentate gyrus (DG) [31] and excitatory neurons in the entorhinal cortex [20]. A previous study indicated that in the entorhinal cortex and prefrontal cortex, pathological tau accumulation occurs predominantly in excitatory neurons rather than in inhibitory neurons [51]. However, we did not detect this tendency in vCA1, which could be attributed to variations across subregions and/or different stages of tau pathology progression.

An early decline in episodic memory is detectable in preclinical AD [52, 53] and is associated with tau pathology [54, 55]. Through specific overexpression of hTau in distinct vCA1 neuron types, we established a direct link between the accumulation of hTau in the PV and excitatory neurons and deficits in social memory. We found that vCA1-hTau^CaMKII^ mice exhibited normal sociability but failed to habituate to the familiar animal and discriminate the familiar mouse from the novel one, indicating that vCA1-hTau^CaMKII^ accumulation disrupted social memory with familiar conspecifics. For vCA1-hTau^PV^ neurons, overexpressing hTau did not affect social familiarization but significantly decreased social activity in the novel subject, suggesting that tau accumulation within PV neurons may specifically disrupt the identification of the novel subject. This finding is in line with the findings from Liang’s laboratory that acute and chronic blockade of PV neurons in the vCA1 by optogenetics and tetanus toxin impaired the identification of novel conspecifics [33]. Importantly, the present study lacks functional results about females, which should be regarded as a weakness considering that sex is one of the most influential risk factors for AD development. Therefore, future research is warranted to address these limitations appropriately.

There are at least three possible reasons for social memory deficits: (1) failure to encode social information during social familiarization, (2) acquisition of social information without its maintenance, and (3) inadequate or inaccurate retrieval of social information. For vCA1-hTau^CaMKII^ accumulation, insufficient retrieval of social memory with familiar conspecifics may contribute to tau-impaired social memory because tau specifically suppressed familiar conspecific recognition-associated firings of excitatory neurons, and photostimulating excitatory neurons during the retrieval phase rather than the encoding phase efficiently rescued tau-impaired social memory. However, for vCA1-hTau^PV^ accumulation, suppression of novel conspecific recognition-associated firings of PV neurons, reduction of GABA release into excitatory neurons and hyperactivation of excitatory neurons during social investigation with novel mice were clearly detected in vCA1-hTau^PV^ mice. When vCA1-hTau^PV^ mice were approaching a novel mouse, photostimulating PV neurons in the vCA1 significantly improved social discrimination between novel and familiar mice. These data indicate that tau-suppressed PV neurons contribute to untimely retrieval of social memory concerning familiar conspecifics by disinhibiting excitatory neurons, thereby leading to the failure to identify novel social conspecifics. Taken together, insufficient and inopportune memory retrieval, rather than social learning (encoding), are responsible for social memory disorders induced by tau accumulation within excitatory and PV neurons, respectively.

Notably, stimulating vCA1-hTau^PV^ neurons at a low gamma wave frequency (40 Hz) could rescue social memory deficits in vCA1-hTau^PV^ mice. In a previous study, when a pair of familiar mice were used as social targets, photostimulating PV neurons in the vCA1 at 8 Hz strongly enhanced social approach [33]. However, in our present study, when using a novel and a familiar mouse as the targets, there was no increase in investigation time in vCA1-hTau^PV^-ChR2 mice under 8 Hz light stimulation. The lack of beneficial effects of photoactivation at 8 Hz may be attributed to differences in social targets and/or the stimulation power utilized. Although 3xTg AD mice and P301L mice are widely used in AD studies, both excitatory and PV neurons accumulate with mislocated p-Tau at the early stage of the AD-like process. This may limit comparative studies of differences in susceptibility of excitatory and PV neurons to pathological tau. Therefore, in future studies, it is necessary to establish AD models in which excitatory and PV neurons are sequentially involved in tau pathology.

Many studies have shown that intracellular tau accumulation can induce severe neural dysfunction. By combining electrophysiology and in vivo fibre photometry data on calcium transit, we found that hTau accumulation in the vCA1 impaired the excitability of both excitatory and PV neurons. It was previously demonstrated that tau could inhibit neural excitation by disrupting the integrity of the axon initiation region [56]. Our recent studies on hippocampal interneurons (i.e., CA3 [56] and DG [31]) revealed that abnormal protein networks, especially those related to the miR92a-vGAT-GABA pathway, were responsible for the dysregulation of GABA metabolism and the reduction in GABA release. Although these molecular mechanisms may also contribute to the dysfunctions of excitatory and PV neurons along with the progressive accumulation of tau, the specific molecular mechanisms involved and the differences between distinct neuronal types deserve further investigation. Furthermore, although tau is primarily known as a cytoplasmic protein, several studies have detected the presence of tau protein in the extracellular space of the brain [57–59]. Using in vivo microdialysis, Yamada et al. [60] reported an increase in extracellular tau protein levels and neuronal activity. It has been shown that presynaptic glutamate release is sufficient to drive tau release [60]. Nevertheless, our findings indicate the absence of hTau transmission from excitatory neurons (CaMKII^+^) to inhibitory neurons (PV^+^) and the lack of significant effects of hTau transmission on the excitabilities of other neuronal types. These observations may be attributed to the inhibitory impact of intracellular tau accumulation on neuronal activities.

Simultaneously, targeting the activity of both excitatory and PV neurons through optogenetic techniques during social recognition becomes challenging when pathological tau accumulates within these neuron types in vCA1. In such cases, reducing the load of pathological tau in vCA1 microcircuits might present a more promising strategy. Accumulating evidence has suggested that targeting TFEB, an essential regulator of the autophagy-lysosomal pathway, is promising for the treatment of neurodegenerative disorders, including AD [61]. Polito et al. [42] reported that overexpression of TFEB effectively reduced NFT pathology, rescued spatial memory and improved synaptic function in rTg4510 mice.

Previous studies have revealed that UA is a safe [62] and effective treatment for several inflammatory diseases and related experimental models, including Parkinson’s disease [63], multiple sclerosis [64], arthritis [65], diabetes [66], traumatic brain injury [67] and high-fat diet-induced cognitive impairments [68]. However, the neuroprotective effects of UA in AD and the detailed underlying mechanisms have not been fully characterized. In the present study, we found that UA could significantly activate TFEB and markedly reduce the pathological tau load in vCA1 of vCA1-hTau mice. hTau-induced social memory loss was also efficiently reversed after 1 month of UA administration in a TFEB-dependent manner. This is the first study to reveal that UA can activate TFEB, reduce pathological tau protein levels and restore microcircuits in vCA1. Given its ability to cross the blood‒brain barrier [69], UA could be a potent TFEB activator with promise for the prevention or treatment of tau pathology in AD patients. Further investigation is warranted to elucidate the mechanisms through which UA activates TFEB, including its potential inhibitory effects on the Akt/mTOR pathway [70]. Additionally, other forms of UA, such as UA liposomes, which have received approval from the State Food and Drug Administration (SFDA) of China for clinical trial evaluation of safety in patients with tumours [71–73] (Clinical Trial Registration No. 2009L00634), should be explored in greater detail.

## Conclusions

We demonstrated distinct protein networks associated with the vulnerability of hippocampal vCA1 to tau pathology. Tau accumulation within excitatory and PV neurons leads to impairments in the recognition of familiar conspecifics and identification of novel conspecifics, respectively. Furthermore, tau accumulation in PV neurons disinhibited local excitatory neurons via insufficient GABA release, specifically during novel conspecific identification. Not only photostimulation of excitatory and PV neurons but also UA treatment efficiently alleviated AD-like social memory loss. Therefore, the present study highlights the vulnerability of the ventral hippocampus and its distinct neurons to tau accumulation, and elucidates the microcircuit mechanism underlying tau-impaired social memory. Furthermore, these novel findings regarding the efficiency of UA and the crucial role of TFEB in tau reduction may provide a promising strategy for future AD treatment.

### Supplementary Information

**Additional file 1: Materials and methods. Table S1** Virus strains and their applications. Table S2. Antibody list. **Fig. S1** A realistic picture for the social memory test. **Fig. S2** Schematic representation of the dissection of vCA1 and dCA1. **Fig. S3** Prominent accumulation of hyper-phosphorylated tau (p-Tau, AT8) in the ventral hippocampal CA1 (vCA1) of 3xTg-AD mice. **Fig. S4** Accumulation of hyper-phosphorylated tau (p-Tau) in the vCA1 of 3-month-old female P301L and 8-month-old male P301S mice. **Fig. S5** Differential proteins detected in the ventral hippocampal CA1 (vCA1) and dorsal hippocampal CA1 (dCA1) in WT and P301L mice. **Fig. S6** Number of differentially phosphorylated proteins/sites detected in the ventral hippocampal CA1 (vCA1) and dorsal hippocampal CA1 (dCA1) in WT and P301L mice. **Fig. S7** Male 3xTg-AD and P301L mice displayed social memory deficits. **Fig. S8** Injection sites of AAV-hSyn-hTau-eGFP virus into the vCA1. **Fig. S9** Overexpression hTau in vCA1 has no effect on anxiety-like behaviors. **Fig. S10** Tau accumulation in the dCA1 has no effect on social memory. **Fig. S11** Hyper-phosphorylated tau (p-Tau) is accumulated in excitatory and PV neurons of 3xTg-AD mice. **Fig. S12** hTau accumulation increases the action potential threshold of CaMKII+ and PV+ neurons in the vCA1. **Fig. S13** No transmission of hTau occurs from excitatory neurons (CaMKII+) to PV neurons in vCA1-hTauCaMKII mice. **Fig. S14** hTau overexpression in PV or excitatory neurons does not affect the electrophysiological properties of excitatory or PV neurons. **Fig. S15** Quantitative analysis of co-location of GCaMP6f and hTau in excitatory and PV neurons. **Fig. S16** Tau accumulation has no effects on eGFP signals of CaMKII and PV neurons during bouts of social interaction. **Fig. S17** Accumulation of vCA1-hTauPV disinhibits excitatory neurons during novel conspecific identification. **Fig. S18** Accumulation of vCA1-hTauPV has no effect on eGFP signals of CaMKII neurons during bouts of social interaction. **Fig. S19** ChR2 is highly expressed in excitatory and PV neurons. **Fig. S20** Photostimulation of excitatory neurons during social exploration has no improvement on social discrimination in vCA1-hTauCaMKII mice. **Fig. S21** Photoactivation of excitatory and PV neurons expressing eYFP had no effects on tau-impaired social memory. **Fig. S22** Photoactivation of excitatory neurons in physical condition at different rhythm has no improvements on social memory. **Fig. S23** Photoactivation of PV neurons in physical condition at different rhythm has no improvements on social memory. **Fig. S24** Ursolic acid (UA) at 30 μmol/L effectively reduces pathological tau without changing tau mRNA. **Fig. S25** CCK-8 assay on cytotoxicity of UA in HEK293-hTau cells. **Fig. S26** Low-dose UA treatment remarkably reduces tau load and ameliorates social memory deficits in vCA1-tau mice. **Fig. S27** Low-dose UA treatment has no toxic effect on social memory in C57BL/6 mice. **Fig. S28** Low-dose UA treatment improves the excitability of excitatory and PV neurons in vCA1-hTau mice.

## Data Availability

All the data from the current study are shown in the manuscript and supplemental materials and are available from the corresponding author upon reasonable request. The raw proteomics data were uploaded to a publicly accessible database, and the ProteomeXchange ID is PXD043883 (please visit the following websites: https://proteomecentral.proteomexchange.org/cgi/GetDataset).

## Abbreviations

AAV: Adeno-associated virus
AD: Alzheimer’s disease
AP: Anteroposterior
dCA1: Dorsal hippocampal CA1
CaMKII: Calcium/calmodulin-dependent protein kinase II
ChR2: Channelrhodopsin-2
Cm: Capacitance membrane
DV: Dorsoventral
eGFP: Enhanced green fluorescent protein
EC: Entorhinal cortex
FC: Fold change
GO: Gene Ontology
GABA: γ-Aminobutyric acid
GCaMP6f: Genetically encoded calcium indicator
hTau: Human tau
iGABASnFR: Intensity-based GABA-sensing fluorescence reporters
LC3B: Microtubule-associated proteins 1A/1B light chain 3B
MAPT: Microtubule-associated protein tau
ML: Mediolateral
MS: Mass spectrometry
NFT: Neurofibrillary tangle
nTg: Nontransgenic
PCR: Polymerase chain reaction
p-Tau: Phosphorylated tau
PV: Parvalbumin
RMP: Resting membrane potential
TFEB: Transcription factor EB
UA: Ursolic acid
vCA1: Ventral hippocampal CA1
