# Supplementary material for: Targeting vulnerable microcircuits in the ventral hippocampus of male transgenic mice to rescue Alzheimer-like social memory loss
Source: Mil Med Res. 2024 Mar 11;11:16. doi: 10.1186/s40779-024-00512-z (PMC10926584; doi:10.1186/s40779-024-00512-z)
Supplement: Supplementary file 1 — Additional file 1: Materials and methods. Table S1 Virus strains and their applications. Table S2. Antibody list. Fig. S1 A realistic picture for the social memory test. Fig. S2 Schematic representation of the dissection of vCA1 and dCA1. Fig. S3 Prominent accumulation of hyper-phosphorylated tau (p-Tau, AT8) in the ventral hippocampal CA1 (vCA1) of 3xTg-AD mice. Fig. S4 Accumulation of hyper-phosphorylated tau (p-Tau) in the vCA1 of 3-month-old female P301L and 8-month-old male P301S mice. Fig. S5 Differential proteins detected in the ventral hippocampal CA1 (vCA1) and dorsal hippocampal CA1 (dCA1) in WT and P301L mice. Fig. S6 Number of differentially phosphorylated proteins/sites detected in the ventral hippocampal CA1 (vCA1) and dorsal hippocampal CA1 (dCA1) in WT and P301L mice. Fig. S7 Male 3xTg-AD and P301L mice displayed social memory deficits. Fig. S8 Injection sites of AAV-hSyn-hTau-eGFP virus into the vCA1. Fig. S9 Overexpression hTau in vCA1 has no effect on anxiety-like behaviors. Fig. S10 Tau accumulation in the dCA1 has no effect on social memory. Fig. S11 Hyper-phosphorylated tau (p-Tau) is accumulated in excitatory and PV neurons of 3xTg-AD mice. Fig. S12 hTau accumulation increases the action potential threshold of CaMKII+ and PV+ neurons in the vCA1. Fig. S13 No transmission of hTau occurs from excitatory neurons (CaMKII+) to PV neurons in vCA1-hTauCaMKII mice. Fig. S14 hTau overexpression in PV or excitatory neurons does not affect the electrophysiological properties of excitatory or PV neurons. Fig. S15 Quantitative analysis of co-location of GCaMP6f and hTau in excitatory and PV neurons. Fig. S16 Tau accumulation has no effects on eGFP signals of CaMKII and PV neurons during bouts of social interaction. Fig. S17 Accumulation of vCA1-hTauPV disinhibits excitatory neurons during novel conspecific identification. Fig. S18 Accumulation of vCA1-hTauPV has no effect on eGFP signals of CaMKII neurons during bouts of social interaction. Fig. S19 ChR2 i [file 40779_2024_512_MOESM1_ESM.docx]

**Materials and methods**

**Tandem mass tag proteomics and phosphoproteomics experiments**

The excised vCA1 and dCA1 tissues were immediately snap-frozen in liquid nitrogen and subsequently ground into a fine powder. The cell powder was then transferred to a 5 ml centrifuge tube. Next, 4 volumes of lysis buffer (8 mol/L urea, 1% protease inhibitor cocktail, 1% phosphatase inhibitor) were added to the cell powder, followed by 3 rounds of sonication on ice using a high-intensity ultrasonic processor (Scientz, China). For the phosphoproteomic experiments, 1% phosphatase inhibitor for phosphorylation was added to the lysis buffer. The resulting mixture was centrifuged at 12,000 *g* at 4 °C for 10 min to remove debris. The supernatant was collected, and the protein concentration was determined using a BCA kit according to the manufacturer’s instructions. After digestion, the tryptic peptides were dissolved in 0.5 mol/L triethylammonium bicarbonate buffer. The peptide samples were labelled with the corresponding tandem mass tag reagent according to the manufacturer’s protocol (Thermo Fisher Scientific, USA) and incubated at room temperature for 2 h. Five microlitres of each sample was pooled, desalted, and analysed by mass spectrometry (MS) to assess labelling efficiency. Following the labelling efficiency check, the samples were quenched by the addition of 5% hydroxylamine. The pooled samples were desalted using a Strata X C18 SPE column (Phenomenex, USA) and dried using vacuum centrifugation. For phosphoproteomic experiments, the peptide mixtures were incubated with an immobilized metal ion affinity chromatography microsphere suspension in loading buffer (50% acetonitrile and 0.5% acetic acid) with vibration. Nonspecifically adsorbed peptides were removed by sequentially washing the ion affinity chromatography microspheres with buffer A (50% acetonitrile and 0.5% acetic acid) and buffer B (30% acetonitrile and 0.1% trifluoroacetic acid). Enriched phosphopeptides were eluted using elution buffer containing 10% NH_4_OH and collected through vibration. The supernatant containing the phosphopeptides was lyophilized for LC-MS/MS analysis. Next, tryptic peptides were dissolved in solvent A (0.1% formic acid, 2% acetonitrile in water) and directly loaded onto a custom-made reversed-phase analytical column (25 cm length, 100 μm i.d.).

The proteomic peptides and supernatant containing phosphopeptides were lyophilized for LC-MS/MS analysis. Next, tryptic peptides were dissolved in solvent A (0.1% formic acid, 2% acetonitrile in water) and directly loaded onto a custom-made reversed-phase analytical column (25 cm length, 100 μm i.d.). Phosphopeptides were separated with a gradient from 8% to 11% solvent B (0.1% formic acid in acetonitrile) over 4 min and then from 11% to 32% in 48 min, followed by a climb to 80% in 4 min; the concentrations were held at 80% for the last 4 min, all at a constant flow rate of 500 nl/min, using an EASY-nLC 1200 UPLC system (Thermo Fisher Scientific, USA). Proteomic peptides were separated with a gradient from 7% to 10% solvent B (0.1% formic acid in acetonitrile) for 4 min and then from 10% to 32% for 49 min, followed by a climb to 80% in 4 min. The concentrations were held at 80% for the last 3 min, all at a constant flow rate of 500 nl/min, using an EASY-nLC 1200 UPLC system (Thermo Fisher Scientific, USA). The separated peptides were analysed using an Exploris480 mass spectrometer (Thermo Fisher Scientific, USA) equipped with a nanoelectrospray ionization source. The resulting MS/MS data were processed using the MaxQuant search engine (v.1.6.15.0, China). To minimize false-positive identifications, a false discovery rate < 1% was required for all the reported proteins. Finally, bioinformatics data analyses were performed using Microsoft Excel (China) and R statistical computing software (China). Fisher’s exact test was used to determine the significance of the gene ontology (GO) enrichment of the differentially expressed proteins, with a *P*-value < 0.05 considered to indicate statistical significance.

For the phosphoproteomic analysis, we defined the hyperphosphorylated and hypophosphorylated proteins as follows. The intensities of the modified peptides (I) were first standardized and converted into relative quantitative values (R) for each sample. Subsequently, pairwise comparisons were conducted between groups. The fold change (FC) was determined by calculating the ratio of the mean intensities for each protein in the two sample groups. To assess the statistical significance of differences between the groups, we performed Student’s *t* test on the relative quantitative values of each protein. Typically, a *P*-value < 0.05 was considered the threshold for statistical significance. Proteins with a *P*-value < 0.05 and a FC > 1.3 were classified as hyperphosphorylated proteins, while proteins with a *P*-value < 0.05 and a FC < 1.0/1.3 were classified as hypo-phosphorylated proteins.

**Table S1** Virus **s**trains and their applications

| **Virus strains** | **Source** | **Identifier** | **For experiment** |
| --- | --- | --- | --- |
| rAAV-hSyn-eGFP | BrainVTA | Cat# PT-1990 | Behavioural test |
| rAAV-CaMKII-mCherry | BrainVTA | Cat# PT-0108 | Behavioural test |
| rAAV-EF1a-DIO-mCherry | BrainVTA | Cat# PT-0013 | Behavioural test |
| rAAV-CaMKII-GCaMPf | BrainVTA | Cat# PT-0110 | In vivo Ca^2+^ recording |
| rAAV-CaMKII-eGFP | BrainVTA | Cat# PT-0290 | In vivo Ca^2+^ recording |
| rAAV-EF1a-DIO-GCaMPf | BrainVTA | Cat# PT-0106 | In vivo Ca^2+^ recording |
| rAAV-EF1a-DIO-eGFP | BrainVTA | Cat# PT-1103 | In vivo Ca^2+^ recording |
| rAAV-CaMKII-iGABASnFR | Brain Case | Cat# BC-0341 | In vivo GABA singal recording |
| rAAV-CaMKII-hChR2(H134R)-eYFP | BrainVTA | Cat# PT-0296 | In vivo optogenetic manipulation |
| rAAV-CaMKII-eYFP | BrainVTA | Cat# PT-0107 | In vivo optogenetic manipulation |
| rAAV-EF1a-DIO-hChR2(H134R)-eYFP | BrainVTA | Cat# PT-0001 | In vivo optogenetic manipulation |
| rAAV-EF1a-DIO-eYFP | BrainVTA | Cat# PT-0012 | In vivo optogenetic manipulation |
| pAAV-hSyn-shTFEB-eGFP | OBiO Technology | Cat# Y14879 | Behavioural test or Western blotting |
| pAAV-hSyn-shNT-eGFP | OBiO Technology | N/A | Behavioural test or Western blotting |
| pAAV-EF1a-DIO-hTau-mCherry | OBiO Technology | N/A | Behavioural test or in vitro patch-clamp recording |
| pAAV-CaMKII-hTau-mCherry | OBiO Technology | N/A | Behavioural test or in vitro patch-clamp recording |
| pAAV-CaMKII-Cre-mCherry | OBiO Technology | N/A | Behavioural test |
| pAAV-EF1a-DIO-hTau-eGFP | OBiO Technology | N/A | Behavioural test |
| pAAV-hSyn-hTau-eGFP | OBiO Technology | N/A | Behavioural test or Western blotting |
| pLenti-hSyn-MAPT-eGFP-3xFLAG-WPRE | OBiO Technology | N/A | Cell transfection |

*GABA* γ-aminobutyric acid, *N/A* not available, *hTau* human tau, *eGFP* enhanced green fluorescent protein, *eYFP* enhanced yellow fluorescent protein, *MAPT* microtubule-associated protein tau

**Table S2** Antibody list

| **Antibodies** | **Host** | **Source** | **Cat#** | **Dilution** |
| --- | --- | --- | --- | --- |
| CaMKII | Rabbit | CST | 3362 | 1:200 (IHC) |
| PV | Rabbit | Abcam | ab11427 | 1:200 (IHC) |
| pT205 | Rabbit | SAB | 1108-2 | 1:200 (IHC) |
| AT8 | Mouse | Thermo | MN1020 | 1:200 (IHC); 1:1000 (Western blotting) |
| HT7 | Mouse | Thermo | MN1000 | 1:1000 (Western blotting) |
| Tau5 | Mouse | abcam | ab80579 | 1:1000 (Western blotting) |
| LC3B | Mouse | abcam | ab192890 | 1:1000 (Western blotting) |
| p62 | Mouse | abcam | ab280086 | 1:1000 (Western blotting) |
| TFEB | Rabbit | CST | 37785 | 1:200 (IHC); 1:500 (Western blotting) |
| β-actin | Rabbit | abclonal | AC006 | 1:1000 (Western blotting) |
| Goat anti-mouse IgG (H+L) | Goat | Invitrogen | ab228307 | 1:3000 (Western blotting) |
| Goat anti-rabbit IgG (H+L) | Goat | Invitrogen | ab228341 | 1:3000 (Western blotting) |
| Alexa Fluor 488 donkey-anti-rabbit IgG (H+L) | Donkey | Invitrogen | A21206 | 1:500 (IHC) |
| Alexa Fluor 488 donkey-anti-mouse IgG (H+L) | Donkey | Invitrogen | A21202 | 1:500 (IHC) |
| Alexa Fluor 546 donkey-anti-rabbit IgG (H+L) | Donkey | Invitrogen | A10040 | 1:500 (IHC) |

*CaMKII* calcium/calmodulin-dependent protein kinase II, *PV* parvalbumin, *TFEB* transcription factor EB, *IHC* immunohistochemistry

**Fig. S1** A realistic picture for the social memory test. A familiar mouse and a novel mouse were placed in the opposing cups, and the subject mouse were freely exploring the arena

**Fig. S2** Schematic representation of the dissection of vCA1 and dCA1. The red regions correspond to vCA1 (**a**) and dCA1 (**b**), respectively. The dissected part is indicated by the red line. dCA1 dorsal hippocampal CA1, vCA1 ventral hippocampal CA1

**Fig. S3** Prominent accumulation of hyper-phosphorylated tau (p-Tau, AT8) in the ventral hippocampal CA1 (vCA1) of 3xTg-AD mice. p-Tau was undetectable in the dorsal hippocampal CA1 (dCA1) of 3xTg-AD mice. Scale bar = 500 μm (vHP), 200 μm (dHP) or 50 μm (vCA1 and dCA1). nTg nontransgenic, vHP ventral hippocampal, dHP dorsal hippocampal

**Fig. S4** Accumulation of hyper-phosphorylated tau (p-Tau) in the vCA1 of 3-month-old female P301L and 8-month-old male P301S mice. **a** Representative images showed an abundant of hyper-p-Tau (AT8) in the ventral hippocampal CA1 (vCA1), but not in the dorsal hippocampal CA1 (dCA1) of 3-month-old female P301L mice. **b** Representative images showed a significant accumulation of AT8 in the vCA1, while no such accumulation was observed in the dCA1 of 8-month-old male P301S mice. Scale bar = 500 μm (vHP) or 200 μm (dHP). nTg nontransgenic, WT wild type, vHP ventral hippocampal, dHP dorsal hippocampal, vDG ventral dentate gyrus, dDG dorsal dentate gyrus

**Fig. S5** Differential proteins detected in the ventral hippocampal CA1 (vCA1) and dorsal hippocampal CA1 (dCA1) in WT and P301L mice. **a** Proteomic analysis revealed the numbers of differential proteins between WT and P301L mice in the vCA1 and dCA1. **b** Radar graph depicts differential proteins between vCA1 and dCA1 under physiological conditions and in the presence of AD-like tau pathology. From the outside to the inside, the first circle represents the name of protein. The orange arrow in the second circle represents the sequence of differential proteins, arranged from smallest to largest by *P*-values. The third circle represents the ratio of differential expression change in the comparison groups, i.e., WT^vCA1^ vs. WT^dCA1^ and P301L^vCA1^ vs. P301L^dCA1^ with log_2_ conversion. In this representation, the color pink indicates upregulation, while blue denotes downregulation. The size of the data point corresponds to the magnitude of the difference. The fourth circle represents the average quantitative value of the two groups. WT wild type, MOBP myelin-associated oligodendrocyte basic protein, MARCKSL1 MARCKS-related protein, VAT1L synaptic vesicle membrane protein VAT-1 homolog-like, DAGLA diacylglycerol lipase-alpha, ATP2B4 plasma membrane calcium-transporting ATPase 4, RPLP1 60S acidic ribosomal protein P1, MOG mannosyl-oligosaccharide glucosidase, LY6H lymphocyte antigen 6H, CNIH2 protein cornichon homolog 2, MBP maintenance complex-binding protein, ACTN2 alpha-actinin-2, PPP3R1 protein phosphatase 3 regulatory subunit 1, SEMA7A semaphorin-7A, GAP43 growth associated protein 43, ANXA11 annexin A11, CALB2 calretinin, ATP2B1 plasma membrane calcium-transporting ATPase 1, ITPKA inositol-trisphosphate 3-kinase A, PPP3CA protein phosphatase 3 catalytic subunit alpha, WFS1 wolframin, SIRT2 NAD-dependent protein deacetylase sirtuin-2, SLC8A2 sodium/calcium exchanger 2, ARHGAP12 Rho GTPase-activating protein 12, NEFL neurofilament light polypeptide, ARPC5 actin-related protein 2/3 complex subunit 5, MAPT microtubule-associated protein tau, SLC25A18 mitochondrial glutamate carrier 2, CPNE7 copine-7, YJEFN3 YjeF N-terminal domain-containing protein 3, DPP10 inactive dipeptidyl peptidase 10, MYO1D unconventional myosin-1D, SCG2 secretogranin-2, FBLN5 fibulin-5, PDE1A calcium/calmodulin-dependent 3’,5’-cyclic nucleotide phosphodiesterase 1A, CaMK1d calcium/calmodulin-dependent protein kinase type 1D, NEFH neurofilament heavy polypeptide, IGSF21 immunoglobulin superfamily member 21, CaMKIId calcium/calmodulin-dependent protein kinase type II subunit delta, MOBP myelin-associated oligodendrocyte basic protein, RPH3A rabphilin-3A, NCAM2 neural cell adhesion molecule 2, GLTP glycolipid transfer protein, TTR transthyretin, FC fold change

**Fig. S6** Number of differentially phosphorylated proteins/sites detected in the ventral hippocampal CA1 (vCA1) and dorsal hippocampal CA1 (dCA1) in WT and P301L mice. Phosphoproteomic analysis revealed the numbers of differentially phosphorylated proteins and their corresponding sites between WT and P301L mice in the vCA1 and dCA1. WT wild type

**Fig. S7** Male 3xTg-AD and P301L mice displayed social memory deficits. Behavioural schematic of social exploration test (**a**) and social memory test (**b**). 3xTg-AD mice (**c**), P301L mice (**e**) and their controls displayed a preference for conspecifics over empty cups, with no significant difference between the groups. In social memory test, 3xTg-AD mice (**d**) and P301L mice (**f**) spent a similar amount of time investigating familiar and novel conspecifics. The social discrimination score was lower in 3xTg-AD mice (**d**) and P301L mice (**f**) than their controls. nTg: *n* = 8 mice; 3xTg-AD: *n* = 8 mice; WT: *n* = 10 mice; P301L: *n* = 8 mice. ^**^*P* < 0.01, as determined by two-way ANOVA or two-tailed unpaired *t*-test. Data were presented as mean ± SEM. nTg nontransgenic, WT wild type

**Fig. S8** Injection sites of AAV-hSyn-hTau-eGFP virus into the vCA1. Representative images showed robust eGFP signals, spanning from bregma -3.08 mm to -3.64 mm, within the vCA1 region. Scale bar = 500 μm. AAV adeno-associated virus, hTau human tau, eGFP enhanced green fluorescent protein, vCA1 ventral hippocampal CA1

**Fig. S9** Overexpression hTau in vCA1 has no effect on anxiety-like behaviors. **a** In the elevated plus maze test, vCA1-hTau mice (*n* = 8) exhibited similar time spent in the open arm and probability of entering the open arm compared to the control group (*n* = 10). **b** In the open field test, vCA1-hTau mice spent a similar amount of time in the center zone and traveled comparable total distances as the control group. Two-tailed unpaired *t* test was used for statistical analysis. Data were presented as mean ± SEM. vCA1 ventral hippocampal CA1, hTau human tau

**Fig. S10** Tau accumulation in the dCA1 has no effect on social memory. **a** Representative images displayed the expression of AAV-hSyn-hTau-eGFP virus in the dCA1. Scale bar = 200 μm (dHP) or 50 μm (dCA1). **b** Overexpression of hTau in the dCA1 had no effect on sociability. **c** Overexpression of hTau in the dCA1 had no effects on social memory. There were no significant differences in interaction time and discrimination score between the two groups. Ctrl: *n* = 9; dCA1-hTau: *n* = 10. ^**^*P* < 0.01, as determined by two-way ANOVA or two-tailed unpaired *t* test. Data were presented as mean ± SEM. dCA1 dorsal hippocampal CA1, dHP dorsal hippocampus, AAV adeno-associated virus, hTau human tau, eGFP enhanced green fluorescent protein

**Fig. S11** Hyper-phosphorylated tau (p-Tau) is accumulated in excitatory and PV neurons of 3xTg-AD mice. **a** Representative images showing the co-localization of p-Tau and CaMKII neuron. Quantitative analysis showed that 0% and 84% p-Tau was co-localized with CaMKII^+^ neurons in nTg and 3xTg-AD mice, respectively. **b** Representative images showing the co-localization of p-Tau and PV neuron. Quantitative analysis showed that 46% and 92% PV^+^ neurons were co-localized with p-Tau in nTg and 3xTg-AD mice, respectively. Co-labeled cells are indicated by arrows. Scale bar = 100 μm. nTg nontransgenic, CaMKII calcium/calmodulin-dependent protein kinase II, PV parvalbumin

**Fig. S12** hTau accumulation increases the action potential threshold of CaMKII^+^ and PV^+^ neurons in the vCA1. **a** Action potential threshold of excitatory neurons was significantly increased in vCA1-hTau^CaMKII^ group. **b** No significant difference of the capacitance membrane (Cm) and resting membrane potential (RMP) was detected between control and vCA1-hTau^CaMKII^ groups. Ctrl: *n* = 19 neurons from 4 mice; vCA1-hTau^CaMKII^: *n* = 22 neurons from 5 mice. **c** Action potential threshold of PV neurons were significantly increased in vCA1-hTau^PV^ group. **d** No significant difference of the Cm and RMP was detected between control and vCA1-hTau^PV^ groups. Ctrl: *n* = 21 neurons from 5 mice; vCA1-hTau^PV^: *n* = 24 neurons from 5 mice. ^*^*P* < 0.05, ^**^*P* < 0.01, as determined by two-tailed unpaired *t*-test. Data were presented as mean ± SEM. vCA1 ventral hippocampal CA1, CaMKII calcium/calmodulin-dependent protein kinase II, PV parvalbumin, hTau human tau

**Fig. S13** No transmission of hTau occurs from excitatory neurons (CaMKII^+^) to PV neurons in vCA1-hTau^CaMKII^ mice. The AAV-CaMKII-hTau-mCherry virus was injected into the vCA1 region, resulting in the generation of vCA1-hTau^CaMKII^ mice. Representative image showed an absence of mCherry^+^ signals in the PV^+^ neurons. Scale bar = 100 μm. CaMKII calcium/calmodulin-dependent protein kinase II, PV parvalbumin, AAV adeno-associated virus, hTau human tau

**Fig. S14** hTau overexpression in PV or excitatory neurons does not affect the electrophysiological properties of excitatory or PV neurons. **a, d** The schematic diagram illustrated the configuration of virus injection and patch-clamp recordings in the vCA1 region. **b** There was no difference in the excitability of CaMKII^+^ neurons between the vCA1-hTau^PV^ and vCA1-mCherry^PV^ groups. **c** There was no difference in the capacitance membrane (Cm) and resting membrane potential (RMP) of CaMKII^+^ neurons between vCA1-hTau^PV^ and vCA1-mCherry^PV^ groups. vCA1-mCherry^PV^: *n* = 21 neurons from 4 mice; vCA1-hTau^PV^: *n* = 22 neurons from 4 mice. **e** There was no difference in the excitability of PV neurons between the vCA1-hTau^CaMKII^ and vCA1-mCherry^CaMKII^ groups. **f** There was no difference in the Cm and RMP of PV neurons between vCA1-hTau^CaMKII^ and vCA1-mCherry^CaMKII^ groups. vCA1-mCherry^CaMKII^: *n* = 20 neurons from 4 mice; vCA1-hTau^CaMKII^: *n* = 21 neurons from 4 mice. As determined by two-way repeated ANOVAs or two-tailed unpaired *t*-test. Data were expressed as mean ± SEM. vCA1 ventral hippocampal CA1, CaMKII calcium/calmodulin-dependent protein kinase II, PV parvalbumin, AAV adeno-associated virus, hTau human tau

**Fig. S15** Quantitative analysis of co-location of GCaMP6f and hTau in excitatory and PV neurons. **a** Co-localization analysis revealed that 85% GCaMP6f was co-localized with hTau, and 77% hTau was co-localized with GCaMP6f in the excitatory neurons of vCA1. **b** Co-localization analysis revealed that 93% GCaMP6f was co-localized with hTau, and 77% hTau was co-localized with GCaMp6f in the PV neurons of vCA1. PV parvalbumin, GCaMP6f genetically encoded calcium indicators

**Fig. S16** Tau accumulation has no effects on eGFP signals of CaMKII and PV neurons during bouts of social interaction. **a** Heat maps and per-bout stacked plots of CaMKII-eGFP signals were aligned to the start of each bout of interaction event in control and vCA1-hTau^CaMKII^ groups. **b** Heat maps and per-bout stacked plots of PV-eGFP signals were aligned to the start of each bout of interaction event in control and vCA1-hTau^PV^ groups. *n* = 5 per group. vCA1 ventral hippocampal CA1, CaMKII calcium/calmodulin-dependent protein kinase II, PV parvalbumin, eGFP enhanced green fluorescent protein, hTau human tau

**Fig. S17** Accumulation of vCA1-hTau^PV^ disinhibits excitatory neurons during novel conspecific identification. **a** Heat maps of CaMKII-GABA signals were aligned to the start of each bout of interaction event. Quantitative analysis showed that vCA1-hTau^PV^ accumulation significantly decreased the area under the curve (AUC) of CaMKII-GABA signals. **b** Heat maps of CaMKII-GCaMP6f signals were aligned to the start of each bout of interaction event. The AUC of CaMKII-GCaMP6f signals was increased when vCA1-hTau^PV^ mice were exploring a novel mouse in the social memory test. *n* = 6 per group. ^**^*P* < 0.01, two-way ANOVA. Data were presented as mean ± SEM. vCA1 ventral hippocampal CA1, PV parvalbumin, iGABASnFR intensity-based GABA sensing fluorescence reporters, GCaMP6f genetically encoded calcium indicators, hTau human tau

**Fig. S18** Accumulation of vCA1-hTau^PV^ has no effect on eGFP signals of CaMKII neurons during bouts of social interaction. Heat maps and per-bout stacked plots of CaMKII-eGFP signals from control and vCA1-hTau^PV^ mice were aligned to the start of each bout of interaction event. *n* = 5 per group. vCA1 ventral hippocampal CA1, CaMKII calcium/calmodulin-dependent protein kinase II, PV parvalbumin, hTau human tau

**Fig. S19** ChR2 is highly expressed in excitatory and PV neurons. **a** Representative images showing AAV-CaMKII-ChR2-eYFP virus along with CaMKII^+^ signals. Quantitative analysis showed that 97% of CaMKII^+^ neurons was co-localized with ChR2. **b** Representative images showing AAV-DIO-ChR2-eYFP virus along with PV^+^ signals. Quantitative analysis showed that 94% of PV^+^ neurons was co-localized with ChR2. Arrows indicated co-labeled cells. Scale bar = 100 μm. AAV adeno-associated virus, CaMKII calcium/calmodulin-dependent protein kinase II, PV parvalbumin, ChR2 channelrhodopsins-2, eYFP enhanced yellow fluorescent protein

**Fig. S20** Photostimulation of excitatory neurons during social exploration has no improvement on social discrimination in vCA1-hTau^CaMKII^ mice. **a** Action potential discharge of a vCA1 neuron in response to patterned blue laser light (473 nm; 5, 10, 20, and 40 Hz; 5 ms pulse) recorded by ex vivo current-clamp recording. **b** Schematic of photostimulation during social exploration. **c** Photostimulating excitatory neurons in the vCA1 had no effect on social interaction time with familiar and novel mouse in the social memory test. The discrimination score was identical between vCA1-hTau^CaMKII^-eYFP and vCA1-hTau^CaMKII^-ChR2 mice. *n* = 9 per group. As determined by two-way ANOVA or two-tailed unpaired *t*-test. Data were presented as mean ± SEM. vCA1 ventral hippocampal CA1, CaMKII calcium/calmodulin-dependent protein kinase II, eYFP enhanced yellow fluorescent protein, ChR2 channelrhodopsins-2, hTau human tau

**Fig. S21** Photoactivation of excitatory and PV neurons expressing eYFP had no effects on tau-impaired social memory. **a** In vCA1-hTau^CaMKII^-eYFP mice, when exploring a familiar mouse, photoactivation of excitatory neurons had no beneficial effect on interaction time and discrimination score. *n* = 10 per group. **b** In vCA1-hTau^PV^-eYFP mice, when investigating a novel mouse, photoactivation of PV neurons did not show any improvements on interaction time and discrimination score. *n* = 8 per group. As determined by two-way ANOVA or two-tailed unpaired *t*-test. Data were expressed as mean ± SEM. vCA1 ventral hippocampal CA1, CaMKII calcium/calmodulin-dependent protein kinase II, PV parvalbumin, eYFP enhanced yellow fluorescent protein, hTau human tau

**Fig. S22** Photoactivation of excitatory neurons in physical condition at different rhythm has no improvements on social memory. **a** AAV-CaMKII-ChR2-eYFP was injected into the vCA1 of C57BL/6 mice. **b** Representative images displayed the expression of AAV-CaMKII-ChR2-eYFP virus in the vCA1. **c** Schematic illustration of social behavior task and photostimulation. **d** Photostimulating excitatory neurons in the vCA1 at 8, 20 or 40 Hz had no effect on social interaction time and discrimination score. *n* = 9 per group. ^**^*P* < 0.01, as determined by two-way ANOVA or two-tailed unpaired *t*-test. Data were presented as mean ± SEM. AAV adeno-associated virus, CaMKII calcium/calmodulin-dependent protein kinase II, vHP ventral hippocampal, vCA1 ventral hippocampal CA1, ITI inter-trial interval, eYFP enhanced yellow fluorescent protein, ChR2 channelrhodopsins-2

**Fig. S23** Photoactivation of PV neurons in physical condition at different rhythm has no improvements on social memory. **a** AAV-DIO-ChR2-eYFP was injected into the vCA1 of PV-Cre mice. **b** Representative images displayed the expression of AAV-DIO-ChR2-eYFP virus in the vCA1. **c** Schematic representation of social behavior task and photostimulation. **d** Photostimulating PV neurons in the vCA1 at 8, 20 or 40 Hz had no effect on social interaction time and discrimination score. *n* = 8 per group. ^**^*P* < 0.01, as determined by two-way ANOVA or two-tailed unpaired *t*-test. Data were presented as mean ± SEM. AAV adeno-associated virus, PV parvalbumin, ITI inter-trial interval, eYFP enhanced yellow fluorescent protein, ChR2 channelrhodopsins-2, vHP ventral hippocampal, vCA1 ventral hippocampal CA1

**Fig. S24** Ursolic acid (UA) at 30 μmol/L effectively reduces pathological tau without changing *tau* mRNA. **a** Western blotting showed that UA treatment at 30 μmol/L, rather than 15 μmol/L, dramatically reduced total tau (tau5, HT7) and phosphorylated tau (p-Tau, AT8) in HEK293-hTau cells compared with vehicle group. **b** In HEK293-hTau cells, no difference of *tau* mRNA was detected between the group treated with 30 μmol/L UA and the vehicle group. ^*^*P* < 0.05, ^**^*P* < 0.01, as determined by one-way ANOVA or two-tailed unpaired *t*-test. All data were expressed as mean ± SEM. hTau human tau

**Fig. S25** CCK-8 assay on cytotoxicity of UA in HEK293-hTau cells. Decreased cell viability was significantly detectable in the group treated with 100 μmol/L UA. ^*^*P* < 0.05, ^**^*P* < 0.01, determined by one-way ANOVA. Data were presented as mean ± SEM. UA ursolic acid

**Fig. S26** Low-dose UA treatment remarkably reduces tau load and ameliorates social memory deficits in vCA1-tau mice. **a** UA administration reduced total tau (tau5), human tau (hTau, HT7) and phosphorylated tau (p-Tau, AT8) in vCA1-hTau mice. **b** In social exploration test, neither low nor high doses of UA changed the interaction time with a social partner and an empty cup. **c** In social memory test, low-dose UA effectively increased interaction time with novel mice and social discrimination index in vCA1-hTau mice as compared with high-dose UA. hTau + Vehicle: *n* = 9; hTau + UA (L): *n* = 10; hTau + UA (H): *n* = 10. ^*^*P* < 0.05, ^**^*P* < 0.01, as determined by one-way ANOVA or two-way ANOVA. Data were presented as mean ± SEM. UA ursolic acid, L low-dose, H high-dose

**Fig. S27** Low-dose UA treatment has no toxic effect on social memory in C57BL/6 mice. **a** In the social exploration test, low-dose UA [15 mg/(kg·d)] had no effect on social interaction time with a social partner and an empty cup. **b** In the social memory test, both UA-treated and vehicle group spent more time to interaction with novel conspecifics. No difference was detected in discrimination score between UA-treated and vehicle groups. Vehicle: *n* = 9; UA (L): *n* = 8. ^**^*P* < 0.01, as determined by two-way ANOVA or two-tailed unpaired *t*-test. Data were presented as mean ± SEM. UA ursolic acid, L low-dose

**Fig. S28** Low-dose UA treatment improves the excitability of excitatory and PV neurons in vCA1-hTau mice. **a** Excitability of excitatory neurons with hTau accumulation were significantly increased after UA treatment at low dose. However, the improvement was completely abolished when *TFEB* was knocked down in vivo. No difference was detected between Ctrl + Vehicle and hTau + UA (L) groups when current was injected at 100, 400 and 450 pA. **b** UA treatment and *TFEB* knockdown had no effect on the capacitance membrane (Cm) and resting membrane potential (RMP) of excitatory neurons in the vCA1. Ctrl + Vehicle: *n* = 23 neurons from 4 mice; hTau + Vehicle: *n* = 25 neurons from 5 mice; hTau + UA (L): *n* = 21 neurons from 4 mice; hTau + shTFEB + UA (L): *n* = 23 neurons from 4 mice. **c** Excitability of PV neurons with hTau accumulation were significantly increased after UA treatment at low dose. However, the improvement was completely abolished when *TFEB* was knocked down in vivo*.* **d** UA treatment and *TFEB* knockdown had no effects on the Cm and RMP of PV neurons in the vCA1. Ctrl + Vehicle: *n* = 23 neurons from 5 mice; hTau + Vehicle: *n* = 24 neurons from 5 mice; hTau + UA (L): *n* = 24 neurons from 5 mice; hTau + shTFEB + UA (L): *n* = 24 neurons from 5 mice. ^*^*P* < 0.05, ^**^*P* < 0.01, as determined by one-way ANOVA or two-way repeated ANOVA. Data were presented as mean ± SEM. UA ursolic acid, L low-dose, hTau human tau, TFEB transcription factor EB
